# The Secreted Proteins of *Achlya hypogyna* and *Thraustotheca clavata* Identify the Ancestral Oomycete Secretome and Reveal Gene Acquisitions by Horizontal Gene Transfer

**DOI:** 10.1093/gbe/evu276

**Published:** 2014-12-18

**Authors:** Ian Misner, Nic Blouin, Guy Leonard, Thomas A. Richards, Christopher E. Lane

**Affiliations:** ^1^Department of Biological Sciences, The University of Rhode Island; ^2^Department of Biological Sciences, The University of Maryland, College Park; ^3^Biosciences, University of Exeter, United Kingdom; ^4^Integrated Microbial Biodiversity Program, Canadian Institute for Advanced Research, Toronto, Ontario, Canada

**Keywords:** oomycete, horizontal gene transfer, evolution, comparative genomics, osmotrophy

## Abstract

Saprotrophic and parasitic microorganisms secrete proteins into the environment to breakdown macromolecules and obtain nutrients. The molecules secreted are collectively termed the “secretome” and the composition and function of this set of proteins varies depending on the ecology, life cycle, and environment of an organism. Beyond the function of nutrient acquisition, parasitic lineages must also secrete molecules to manipulate their host. Here, we use a combination of *de novo* genome and transcriptome sequencing and bioinformatic identification of signal peptides to identify the putative secreted proteome of two oomycetes, the facultative parasite *Achlya hypogyna* and free-living *Thraustotheca clavata*. By comparing the secretomes of these saprolegnialean oomycetes with that of eight other oomycetes, we were able to characterize the evolution of this protein set across the oomycete clade. These species span the last common ancestor of the two major oomycete families allowing us to identify the ancestral secretome. This putative ancestral secretome consists of at least 84 gene families. Only 11 of these gene families are conserved across all 10 secretomes analyzed and the two major branches in the oomycete radiation. Notably, we have identified expressed elicitin-like effector genes in the saprotrophic decomposer, *T*. *clavata*. Phylogenetic analyses show six novel horizontal gene transfers to the oomycete secretome from bacterial and fungal donor lineages, four of which are specific to the Saprolegnialeans. Comparisons between free-living and pathogenic taxa highlight the functional changes of oomycete secretomes associated with shifts in lifestyle.

## Introduction

The Oomycota are a diverse assemblage of fungal-like protists placed within the eukaryotic supergroup, Stramenoplia (Dick et al. 1999; Dick 2001; Adl et al. 2012) Oomycetes are the most diverse lineage of heterotrophic stramenopiles currently described (Dick 2001) and are ubiquitous members of freshwater aquatic communities and damp terrestrial environments (with fewer species inhabiting estuarine or marine environments). In their vegetative state, oomycetes take the form of colorless, coenocytic (multinucleate) siphons. Although many oomycetes are saprobes, it is the numerous parasitic members of the phylum that garner the most attention. These parasitic oomycetes infect a diverse array of plants, invertebrates, and vertebrates (Seymour and Johnson 1973; Noga and Dykstra 1986; Mendoza et al. 1993; Hussein et al. 2001; Latijnhouwers et al. 2003). Plant and animal infections range from those that are chronic but nonlethal to those that are highly virulent and destroy the host organism. Oomycete infections of plants can cause blights, wilts, cankers, rusts, lesions, and/or (root) rots, contributing to billions of US dollars worth of agricultural losses each year (Tyler 2001; Nowicki et al. 2012).

Like many species that survive via dependent interaction on other organisms (dead or alive), osmotrophic oomycetes secrete a suite of proteins with functions including basic metabolic processes, nutrient acquisition, cell wall manufacture/adhesion/digestion, and virulence (Kamoun 2009; Raffaele et al. 2010; Brown et al. 2012).

Collectively termed the “secretome,” these proteins occur across a wide taxonomic range of osmotrophs, including fungi, oomycetes, and bacteria (Choi et al. 2010) often reflecting the niche these microbes reside in, rather than there phylogenetic affinities (Soanes et al. 2008). The plant pathogenic oomycetes in the genera *Phytophthora* and *Hyaloperonospora* have characteristic sequence motifs, RxLR and LFLAK-DEER (Crinklers), present across hundreds of secreted proteins. This specialized set of effector proteins is responsible for suppression of host defense and is capable of triggering (necrotrophs) or preventing (hemibiotrophs) localized cell death in their host, among other pathogenic effects (Bos 2007; Birch et al. 2008, 2009; Cheung et al. 2008; Levesque et al. 2010; Oh et al. 2010; Stassen and Van den Ackerveken 2011).

Elicitins and elicitin-like proteins, which trigger the hypersensitive response in the host plants (Jiang, Tyler, Whisson, et al. 2006), are a class of effector proteins responsible for extracellular lipid transport that were believed to be exclusive to *Phytophthora* and *Pythium* (Panabieres et al. 1997; Jiang, Tyler, Whisson, et al. 2006) but recently identified in the *Saprolegnia parasitica* genome (Jiang et al. 2013). Elicitin-like genes are highly divergent, but appear to have functions related to true Elicitins. A characteristic feature of this gene family is the presence of three disulfide bonds, formed from six cysteine residues, necessary to stabilize the alpha-helix (Fefeu et al. 1997; Boissy et al. 1999).

In the oomycetes secretome proteins are often encoded by genes located in “labile” and variable regions of the genome, usually flanked by transposable elements and chromosomal regions with high cross over rates (Raffaele et al. 2010). These regions are typified by lower levels of genome conservation and therefore demonstrate higher rates of gene duplication and accelerated rates of sequence evolution, leading to protein divergence and neofunctionalization (Jiang et al. 2008; Soanes and Talbot 2008; Raffaele et al. 2010; Raffaele and Kamoun 2012). The increased evolutionary rate identified in oomycete effector gene families has therefore been suggested to facilitate host jumps and adaptation to novel host defense systems, making oomycetes highly successful pathogens (Raffaele and Kamoun 2012). Understanding the evolution of the oomycete secretome is therefore important, because it encompasses the proteins that drive interactions between parasite and the host environment.

Comparative studies of plant parasitic fungi and oomycetes in the Peronosporaleans show similarities in secretome composition and function (Brown et al. 2012). These similarities are assumed to be the result of convergent evolution. However, Richards et al. (2011) examined the effect of fungal derived horizontal gene transfer (HGT) on the genomes of *Phytophthora ramorum*, *Phytophthora sojae*, *Phytophthora infestans*, and *Hyaloperonospora parasitica* and were able to identify 34 candidate HGTs. Seventeen of these gene families encode proteins that function as part of the secretome, and many have undergone large-scale expansion by gene duplication post transfer, implicating HGT as an important evolutionary mechanism in the oomycetes. Similarly, Belbahri et al. (2008) have identified a bacterial-derived HGT of a candidate plant virulence factor, a cutinase gene family homolog, into the oomycete lineage.

Members of the Saprolegnialeans, which can infect fish (Burr and Beakes 1994; Hussein et al. 2001; van West 2006; Sosa et al. 2007; Oidtmann et al. 2008; Ke et al. 2009), decapods (Unestam 1965; Cerenius and Söderhäll 1984; Oidtmann et al. 2004), and even some plants (Madoui et al. 2009; Trapphoff et al. 2009), have been understudied relative to the agriculturally relevant members of the Peronosporaleans. Moreover, comparisons between closely related nonpathogenic saprobes and the pathogenic oomycetes are lacking, due to the absence of genomic data from the nonpathogenic forms. The recent release of the *S. parasitica* genome (Jiang et al. 2013) has provided genomic data from the Saprolegnialeans from a facultative pathogen of fish. For comparative purposes, we identified two saprolegnian species for genome sequencing: the facultative decapod parasite *Achlya hypogyna* (PRJNA169234) and the nonpathogenic saprobe *Thraustotheca clavata* (PRJNA169235). These organisms provide an opportunity to understand the evolution of the secretome relative to lifestyle and across the deepest division in the oomycetes. We used a bioinformatic approach (Kamoun 2009; Choi et al. 2010; Raffaele et al. 2010; Brown et al. 2012) to identify the proteins belonging to the secretomes of *Ac. hypogyna* and *T*. *clavata*. The secretome proteins were categorized into orthologous gene families and compared with the secretomes from the Peronosporalean and Saprolegnialeans oomycetes and the Hyphochytridiomycete, *Hyphochytrium catenoides* (Richards et al. 2011), the putative sister group of the oomycetes (Van der Auwera et al. 1995; Levesque et al. 2010; Raffaele et al. 2010; Links et al. 2011).

Although the complete genomes of *Ac. hypogyna* and *T*. *clavata* have additional interesting features, here we focus on the secretome of these organisms and what the expansion of available Saprolegnean genomes can reveal about this critical gene set, across oomycetes. We were able to 1) identify and annotate the ancestral secretome of the two most diverse oomycete clades; 2) compare the Saprolegnialean-specific secretome with the secretome of the Peronosporaleans, highlighting the differences in metabolic and pathogenic genes; 3) characterize the evolution of secretome genes and functions across four distinct lifestyles; and 4) identify novel HGT events key to the evolution of the Saprolegnialean secretome.

## Materials and Methods

### Culturing and Nucleotide Extraction

*Achlya hypogyna* (ATTC 48635) and *T*. *clavata* (ATCC 34112) were obtained from American Type Culture Collection (Manassas, VA). Cultures were maintained on cornmeal agar plates and grown in 1.5 l of 50% Luria Broth (LB) at 25 °C for extraction. After 5 days 4.5 l of each species in 50% LB were flash frozen with liquid nitrogen before being ground with mortar and pestle into a powder. While frozen, 5 ml of ground material was placed into 30 ml of Carlson Lysis Buffer (Carlson et al. 1991) with 0.1 mg/ml of Proteinase K. Samples were incubated at 65 °C for 60 min followed by 20 min on ice. Samples were centrifuged at 7,130 ×g for 10 min to remove cellular debris, supernatant was combined with 20 ml of phenol:chloroform:isoamyl alcohol (25:24:1) and gently mixed for 5 min. Extraction was centrifuged at 7,130 ×g for 5 min. The phenol extraction was repeated twice. Nucleic acid was precipitated using 30 ml of 0 °C 95% EtOH and 1/10 volume of 3M sodium acetate, and samples were placed in a freezer overnight (−20°C). The following day samples were centrifuged at 7,130 rpm for 30 min to pellet nucleic acids. Pellets were washed twice with 70% EtOH and resuspended in 2 ml of TE. Samples were treated with RNAse A (10 mg/ml), incubated at 37 °C for 60 min, and precipitated as above. DNA quantity and quality was accessed using a NanoDrop 8000 (Thermo Scientific, Wilmington, DE). Samples were stored at −20 °C.

For RNA extraction, cultures of *Ac. hypogyna* and *T*. *clavata* were acclimatized at 25 °C for 2 weeks at 12:12 Light:Dark before 16 LB 50% concentration and 16 diH_2_O 150 ml subcultures were created, each with three autoclaved hemp seeds. Cultures were then incubated at 4, 15, 25, and 35 °C and harvested at 0.5, 1, 3, and 6 h in both light and dark conditions to collect the widest spectrum of RNA possible. Fresh material was ground under liquid nitrogen immediately after harvest, and ground material was placed directly in extraction buffer. RNA was extracted from *Ac. hypogyna* using the Qiagen RNeasy kit (Qiagen, CA) according to the manufacturer’s protocol. RNA from all experiments was pooled and quality was assessed using a NanoDrop 8000 (Thermo Scientific, CA).

### Sequencing and Assembly

#### Genome

*Achlya hypogyna* DNA was sequenced using one half plate of Roche 454 FLX (Margulies et al. 2005) and one lane of 72 cycle paired-end Illumina-Solexa (Bentley et al. 2008). Roche 454 reads were trimmed on the CLC Genomics Workbench v5.0 (CLC Bio Aarhus, Denmark) using the default settings. Illumina reads were trimmed using CLC with default setting while removing sequences shorter than 70 bp. The 454 trimmed reads were combined with the trimmed Illumina reads for de novo assembly on the CLC Genomics Workbench. Default settings were used for de novo assembly with the length fraction set to 0.7 and the similarity set to 0.9, the minimum distance for paired-end Illumina reads was 150 bp with the maximum distance set to 217 bp.

*Thraustotheca clavata* DNA was sequenced using one half plate of 3 kb paired-end Roche 454 FLX and two lanes of 108 cycle paired-end Illumina. Roche 454 reads were trimmed on the CLC Genomics Workbench v5.0 using the default settings, we removed 12 bases from the 5′-end and reads lengths that were under 150 bp and over 450 bp. Illumina reads were trimmed using CLC using default setting while removing sequences shorter than 40 and 9 bp from the 5′-end. Length and 5′-sequence removal thresholds were determined by analyzing nucleotide frequency plots generated using FastQC v0.10.0 (http://www.bioinformatics.babraham.ac.uk/projects/fastqc/, last accessed December 18, 2014). The 454 trimmed reads were combined with the trimmed Illumina reads for de novo assembly on the CLC Genomics Workbench. Default settings were used for de novo assembly with the length fraction set to 0.3 and the similarity set to 0.95 for 454 reads and 0.99 for Illumina, the minimum distance for paired-end 454 reads was 300 bp with the maximum distance at 3,500 bp, for Illumina reads the minimum was 179 bp and the maximum at 716 bp.

#### Transcriptome

Library construction and sequencing via the Illumina GAII were performed by Genome Quebec using one lane of single 108 bp reads. The data were trimmed on CLC Genomics Workbench (v5.0) to remove reads shorter than 70 bp and those reads whose cumulative bases with quality scores below 0.05. Default parameters for transcriptome assembly were used to create assemblies in CLC.

### Identification and Annotation of Secretome Proteins

The *Ac. hypogyna* and *T*. *clavata* proteins were identified using MAKER v2.31.4 program (Cantarel et al. 2008) MAKER was trained using the protein sequences from *S*. *parasitica* (Broad Institute) and transcripts from *Ac. hypogyna* or *T*. *clavata*, respectively (Misner et al. 2013). The MAKER pipeline was set to create *ab initio* gene predictions using repeat masked and unmasked genomic data using SNAP (Korf 2004) and GeneMark (Lomsadze et al. 2005) with the annotation edit distance = 0.85. SNAP was also used to make HMM-based gene predictions as outlined in the MAKER manual.

To identify proteins belonging to the secretomes of all ten oomycetes and the one hyphochytridiomycete analysed in this study, the predicted proteomes of each species were analyzed by four bioinformatic tools, SignalP v4.0 (Emanuelsson et al. 2007), TargetP v1.1 (Emanuelsson et al. 2000), WoLF PSORT (Horton et al. 2007), and TMHMM v2.0 (Sonnhammer et al. 1998). For a protein to be included in the putative secretome it must have a SignalP *D*-score ≥0.5, a localization of “S” in TargetP, a WoLF PSORT annotation of extracellular, and no transmembrane regions after the signal peptide. These programs were run successively, in the order above, removing failed proteins at each step. TargetP was run using the “nonplant” setting and WoLF PSORT was run with the “fungi” setting as in (Raffaele et al. 2010).

Complete protein sets from all 11 taxa analysed were placed into gene families using OrthoMCL (Chen et al. 2006, 2007). Functional annotations were made by running PFAM (Punta et al. 2012) on the CLC Genomics Workbench v5.1 (CLC Bio Aarhus, Denmark), BlAST2GO (Conesa et al. 2005) and Reversed Position Specific (RPS)-BLAST against the KOG-LE database (ftp://ftp.ncbi.nih.gov/pub/mmdb/cdd/, last accessed December 18, 2014) with a cutoff of 1e^−^^5^. Each OrthoMCL group in this study was assigned a KOG category based upon the majority consensus for the group. For consistency, we predicted the secretome data sets for all 11 organisms using the same methodology and provide the sequences for these data sets (supplementary file S1, Supplementary Material online). Our secretome data were then combined with the secretome predictions from the other available crown oomycetes and *Hyp*. *catenoides*, in order to identify shared, unique, and overrepresented gene families. Carbohydrate-active (CAZy) enzymes were annotated by submitting proteins from each secretome to the dbCAN webserver (Yin et al. 2012) available at http://csbl.bmb.uga.edu/dbCAN/annotate.php (last accessed December 18, 2014). To validate gene gains and losses “missing” secretome genes were BLAST searched against the full genome from each organism using tBLASTn. Protein families that were “lost” from the secretome but identified in the cytosolic proteome were manually inspected for a proper start codon to minimize false negative N-terminal signal peptide predictions.

To verify protein expression *Achlya hypogyna* and *T*. *clavata* secretome proteins were reciprocal BLAST searched against their respective transcriptome assemblies using BLAST + (v2.2.28). Transcriptome nucleotide sequences were compared with secretome protein databases using BLASTx with an *e*-value of 1e^−^^10^. Secretome proteins were compared with transcriptome nucleotide databases using tBLASTn and an *e*-value of 1e^−^^10^. Reciprocal BLAST hits were determined using a custom python script.

### Secretome Evolution Analysis

Secretome proteins were assigned to 886 OrthoMCL gene families and 3,348 were unassigned genes (supplementary file S1, Supplementary Material online). Individual gene families contained both secreted and nonsecreted protein members. These were aligned in MAFFT v7.017 (LINSI method), and gene trees were generated using RAxML using the PROTCATLG model with 500 bootstraps (100 bootstraps for families with more than 200 sequences). Individual gene family trees were reconciled to the species tree was scored for gains, duplications, and losses with NOTUNG v2.8.1.2 (Durand et al. 2006; Vernot et al. 2008; Stolzer et al. 2012). NOTUNG (1.5 duplication and 1 loss cost) was first run in batch mode and trees were rearranged using a “threshold” of 80%. Rearranged trees were reconciled against the species tree using the “phylogenomics” flag. Singletons and unassigned genes were scored as novel gains in each of the leaf nodes where they occurred. The species tree used to reconcile individual gene trees was generated via RAxML 8.0.22 (PROTOGAMMALG model, 1,000 bootstraps) from a concatenated MAFFT (LINSI method) alignment of 15 gene families with a single representative from each taxon. The 15 one to one gene families were identified through an all versus all BLASTp (1e^−^^20^ cut-off).

### Phylogenetic Analysis

Secretome proteins from *Ac. hypogyna* and *T*. *clavata* were clustered into 422 unique gene families using OrthoMCL representing 874 proteins. For each of the 422 OrthoMCL groups the longest protein sequence, from *Ac. hypogyna* or *T*. *clavata*, was identified and extracted using a custom Perl script. In the event of a tie, multiple sequences were extracted for that OrthoMCL group. The 478 longest sequences from each gene family (including ties) were run, individually, through a gene-by-gene phylogenetic pipeline to generate fast maximum-likelihood (ML) trees as in Richards et al. (2011). Briefly, secretome proteins were compared with a database containing 895 (215 eukaryotic and 680 prokaryotic; supplementary file S2, Supplementary Material online) genomes using BLASTp (cutoff of 1e^−^^10^). Similar sequences were aligned using MUSCLE (Edgar 2004a, 2004b). TrimAl (Capella-Gutierrez et al. 2009) was then used to automatically mask and remove regions with ambiguous or “gappy” alignment. FastTree (Price et al. 2010) was used to compute the approximate likelihood ratio test trees from the masked alignments. The generated trees were manually curated to identify HGT, putative function, and gene family composition. For candidate HGTs we refined the alignment and mask manually using Seaview (Gouy et al. 2010), removing distant paralogs, gappy, and partial sequences and checking GenBank nr, GenBank expressed sequence tags (ESTs), and DOE JGI genome databases for additional relevant taxa and sequence diversity. For the final phylogenies we used PhyML (Guindon et al. 2005) with the model parameters estimated for each data set (table 2) using ProtTest 3 (Darriba et al. 2011) and with 500 bootstrap replicates.

### Alternative Tree Topology Tests

For four trees where the phylogeny demonstrated direct support for HGT (i.e., an oomycete group branching with and within a donor group [fungi or bacteria] with strong bootstrap support), we conducted alternative topology tests. To further test, the topological support consistent with HGT we recalculated the best ML tree using RAxML (Stamatakis 2006) constraining monophyly of the donor group (illustrated in supplementary figs. S3 and S7–S9, Supplementary Material online) using the PROTCATWAGF model, and the final ML optimization was done with the +Γ model (not CAT—i.e., PROTGAMMAWAGF). The trees, constrained and unconstrained, for each alignment were combined to a single file and used as input to Consel (Shimodaira and Hasegawa 2001) where they were compared using the approximately unbiased test (Shimodaira 2002; see table 3).

## Results

### Genome Assembly

Using Illumina and Roche 454 sequencing technologies and the CLC Genomics Workbench (v 5.0), we were able to assemble high-quality draft genomes from *Ac*. *hypogyna* and *T*. *clavata* using a combination of Roche 454 and Illumina paired-end sequencing. The *Ac*. *hypogyna* de novo assembly consisted of 3,075 contigs with an average length of 14,124 bp and a N_50_ of 52,035 bp totaling 43.43 Mb of assembled sequence data (table 1). We were able to estimate the completeness, and size, of the genome by using the Conserved Eukaryotic Genes Mapping Approach (CEGMA) and BLAT. The *Ac*. *hypogyna* genome was 98% complete, having 243 of the 248 CEGs. Mapping of transcriptome sequences (10,349 transcripts) to the *Ac*. *hypogyna* genome assembly using BLAT showed that 89.9% of the expressed genes were present in the assembled genome (cutoff of 1e^−^^10^). Based upon the CEGMA and BLAT analyses, the estimated genome size for *Ac*. *hypogyna* is between 44 and 48Mb. The *Ac*. *hypogyna* assembly had a GC content of 56.80% with a 60.71% GC content in the coding regions. Repetitive genomic elements were identified using de novo RepeatScout libraries in RepeatMasker and it was determined that 8.37% of the assembled *Ac*. *hypogyna* DNA was repetitive (table 1).

**Table 1:** Genome Assembly Statistics for Organisms Included in This Study

Genome Statistic	*Achlya hypogyna*	*Thraustotheca clavata*	*Saprolegnia parasitica*	*Phytophthora infestans*	*Albugo candida*	*Pythium ultimum*	*Phytophthora capsici*	*Phytophthora ramorum*	*Phytophthora sojae*	*Hyaloperonospora arabidopsis*
Estimated genome size (Mb)	48	51	63	240	45	45	64	65	95	78.3
Number of contigs	3,062	5,336	4,125	18,288	2,359	1,747	917	2,576	862	3,108
N50 (kb)	52	15	34.5	44.5	77	124	34.6	47.5	390	41.8
Total contig length (Mb)	43.43	39.1	48.1	190	33.9	42.8	56	66.6	82.6	78.3
G + C content (%)	56	38	58	51	43	52	50	54	54	47
Repeat (%)	8	4	17	74	17	7	14	28	39	35
Number of predicted proteins	17,430	12,154	17,065	17,797	15,824	15,291	19,805	15,743	16,989	15,510

The same procedures were used to evaluate the *T*. *clavata* de novo assembly, which consisted of 5,338 contigs having an average length of 7,330 bp and a N_50_ of 15,004 bp totaling 39.1 Mb of assembled DNA. CEGMA analysis found 188 complete CEGs and suggested a 75.81% complete assembly. Contrary to CEGMA, BLAT mapping indicated that 87.94% of the12,841 *T*. *clavata* RNAseq transcripts were present in the assembly. Based upon these analyses the estimated genome size of *T*. *clavata* is between 48 and 51 Mb. The GC content of *T*. *clavata* was 37.92% with 40.33% GC content in coding regions. The repetitive DNA content of *T*. *clavata* was determined to be 4.10% using the same procedures for the *Ac*. *hypogyna* repeat identification (table 1).

### Secretome Identification and Composition

To identify secretome proteins, we used an in silico redundant conservative approach (Torto et al. 2003; Haldar et al. 2006; Win et al. 2006; Kamoun 2009; Brown et al. 2012). Signal peptides were predicted using SignalP v4.0, subcellular targeting was completed using TargetP and WoLF-PSORT, and only proteins predicted as secreted by all three methods were considered to be part of the secretome. Transmembrane domains were identified using TMHMM, and any secreted proteins containing transmembrane domains after the signal peptide were excluded from the secretome. Therefore, the secretome constituents reported here (table 1) are conservative predictions. Given the nature of this analysis incomplete gene prediction, particularly failure to accurately recover the 5′ of the gene model and therefore the N-terminus of the predicted protein, will directly affect the ability to positively identify secretome proteins. However, previous studies have shown that sampling and cross comparing multiple lineages can partially account for this source of error when putatively secreted proteins are compared across orthologue groups (Braaksma et al. 2010).

Each of the 11 secretomes (10 oomycetes + *Hyp. catenoides*) in this study were identified using the above procedure and individually submitted to OrthoMCL (www.orthomcl.org, last accessed December 18, 2014) for gene family assignment to existing OrthoMCL groups (Chen et al. 2006, 2007). Proteins were placed into OrthoMCL groups using BLASTp with an *e*-value of 1e^−^^5^ and 50% match. The *Ac. hypogyna* predicted secretome consisted of 262 gene families, containing 739 individual proteins (table 2). Thirty-eight proteins matched other sequences in the online database but had “no group” (designation by OrthoMCL DB). An additional 218 proteins had no OrthoMCL database hits and are considered unique. The predicted secretome of *T*. *clavata* contained 160 gene families encompassing 405 individual proteins, 25 of which had no group and 52 that had no hits to the OrthoMCL database (table 2). Therefore, although the *T*. *clavata* secretome is smaller, a higher proportion of the *T*. *clavata* secretome (353/405 proteins) had OrthoMCL group assignments than *Ac. hypogyna* (521/739 proteins).

**Table 2:** OrthoMCL Distributions of Secretome Proteins by Species

Species	SAPpar	ACHhyp	THRcla	PHYcap	PHYinf	PHYram	PHYsoj	HYAara	PYTult	ALBcan	HYPcat
Lifestyle	Facultative	Facultative	Saprotroph	Hemibiotroph	Hemibiotroph	Hemibiotroph	Hemibiotroph	Obligate biotroph	Necrotroph	Obligate biotroph	Saprotroph
Total secretome	986	739	405	972	1,131	1,187	3,085	438	679	206	460
Number of hits in OrthoMCL	613	521	353	869	876	1,179	1,303	258	601	107	260
Number of groups	257	262	160	271	282	338	332	147	203	70	181
Number of no Group	46	38	25	55	71	91	112	14	26	6	10
Number of no hits	373	218	52	103	255	8	1,782	180	78	99	200
Proteins with transcript	NA	309	281	NA	NA	NA	NA	NA	NA	NA	NA

Note.—SAPpar, *S. parasitica*; ACHhyp, *Ac*. *hypogyna*; THRcla, *T*. *clavata*; PHYcap, *Ph*. *capsici*; PHYinf, *Ph*. *infestans*; PHYram, *Ph*. *ramorum*; PHYsoj, *Ph*. *soja*e; HYAara, *Hya*. *arabidopsi*s; PYTult, *Py*. *ultimum*; ALBcan, *Al*. *candida*; HYPcat, *Hyp*. *catenoides*.

The *Ac. hypogyna* and *T*. *clavata* secretomes were compared with the secretomes of eight oomycetes, and *Hyp*. *catenoides* using gene family clustering (table 2). The unknown sequences from all 11 secretomes (those that had no hits in OrthoMCL or returned hits with no group) were compared by BLAST against one another to identify potential homology between these nonannotated secretome proteins. BLAST results identified no significant similarity in any of the comparisons (data not shown).

### Ancestral Oomycete Secretome

**Fig. 1.—:**
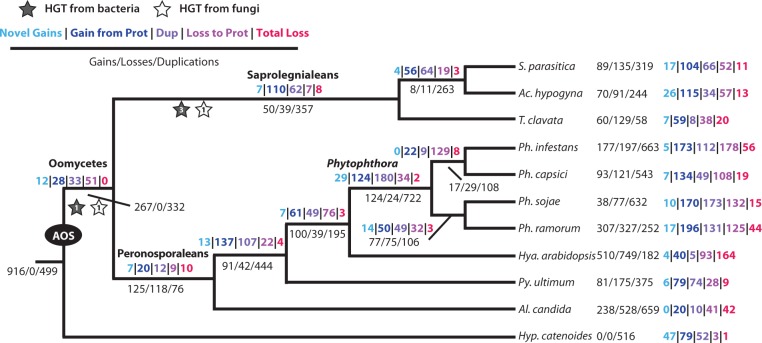
Predicted evolutionary pattern of gene families gained and lost in representative oomycetes. Numbers in black represent gains, duplications, and losses of all gene families. Numbers in color indicate evolutionary events specific to the genes within each gene family predicted to be in the secretome. Stars signify the acquisition of genes via HGT, leading to the Saprolegnialean taxa. A minimum of 84 OrthoMCL gene families are inferred to represent the AOS.

**Fig. 2.—:**
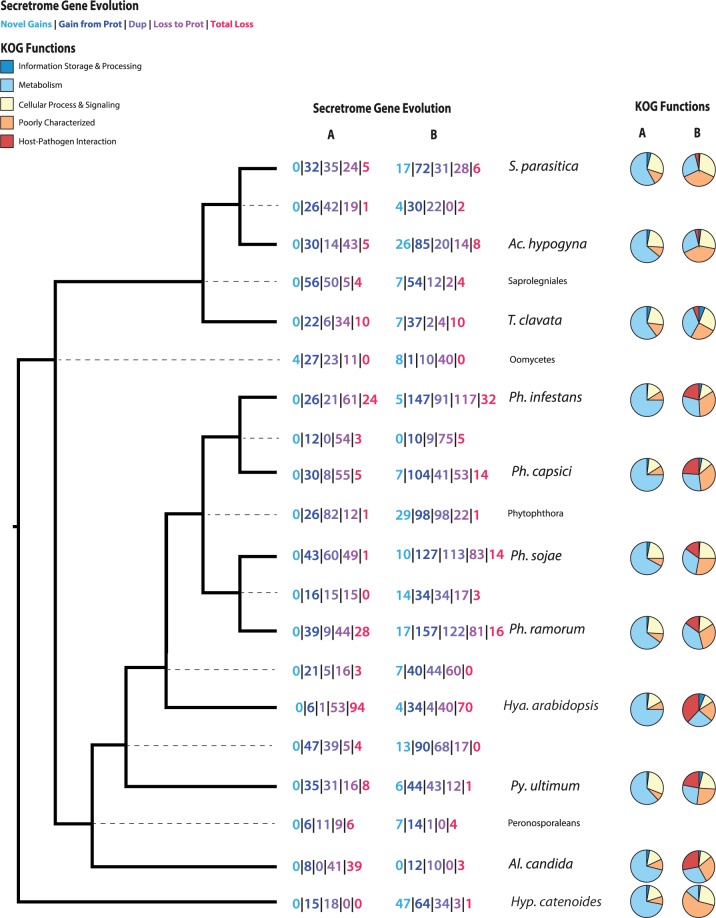
Evolutionary patterns of secretome genes and their inferred KOG function. Column (*A*) under both headings represents the proteins that are derived from the AOS. Column (*B*) includes numbers and functions of secretome proteins not inferred to have been derived from the AOS. AOS-derived proteins are mostly associated with metabolic functions. In contrast, non-AOS derived proteins include the host–pathogen interaction genes and appear to be less conserved, owing to the large proportion that are poorly characterized.

To identify the ancestral oomycete secretome (AOS) we analyzed the three saprolegnian taxa in comparison with seven sequenced members of the Peronosporaleans. Collectively, these species represent a wide range of oomycete lifestyles, from free-living saprobes to obligate parasites of plants (table 2; Haas et al. 2009; Levesque et al. 2010; Raffaele et al. 2010; Links et al. 2011; Jiang et al. 2013). Using the secretome of the putative sister group of the oomycetes, Hyphochytridiomycete, *Hyp*. *catenoides*, for an outgroup we utilized NOTUNG (Durand et al. 2006; Vernot et al. 2008; Stolzer et al. 2012) to model gene gain, loss, and duplication. Based on this comparative analysis, the last common ancestor of the ten oomycetes investigated contained 84 gene families (OrthoMCL groups), which we infer were represented by at least 266 proteins in the AOS (fig. 1). Thirty-eight of the 84 AOS gene families were present in the secretome of *Hyp*. *catenoides* (460 individual proteins). Only 11 of the 84 gene families occur in all ten oomycete secretomes and two of those families are also present in *Hyp*. *catenoides* (supplementary file S1, Supplementary Material online). Additionally, duplication of these proteins represents a considerable factor across the oomycete species tree (fig. 2), demonstrating considerable expansion and reconfiguration of the secretome across the oomycetes. *Achlya hypogyna* encodes 264 proteins derived from the AOS (121 with sequence data supporting mRNA expression), whereas *T*. *clavata* has only 202 AOS-derived proteins (136 with sequence data supporting mRNA expression).

Putative functional annotation of the AOS ranged from amino acid and carbohydrate metabolism, and cell wall degrading enzymes (CWDEs), covering 11 of the 25 KOG categories (supplementary file S1, Supplementary Material online). Twelve of the 84 AOS gene families had no hits to the KOG database or were of “general” or “unknown” function (supplementary table S1, Supplementary Material online). “Carbohydrate transport and metabolism” (KOG: G) represented the largest portion of the AOS, with 31 gene families present, making up 37% of the entire AOS. Moreover, 7 of the 11 gene families that putatively encode secreted proteins found in all oomycetes (64%) were also in KOG category G. “Posttranslational modification, protein turnover, chaperones” (KOG: O) was the next most abundant group, containing 13 gene families, followed by “signal transduction mechanisms” (KOG: T) and “amino acid transport and metabolism” (KOG: E), with nine and six gene families, respectively (supplementary table S1, Supplementary Material online). CAZy enzymes were present in 30 of the 84 AOS gene families, as described below.

Phylogenetic analysis of the AOS showed that 7 secretome gene families were exclusive to oomycetes (supplementary table S1, Supplementary Material online), whereas five families were too divergent to generate alignments (BLASTP cutoff of 1e^−^^10^) for phylogenetic analysis when sampling across our local genome database of 895 genomes, including 215 eukaryotes and 680 prokaryotes (supplementary file S2, Supplementary Material online). BLAST searches of these sequences to the NCBI *nr* database (March 2014) also did not return any low *e*-value (<1e^−^^5^) hits outside of the oomycetes sampled.

### Elicitins

Eight highly divergent gene families in our analysis were predicted to encode elicitin-like proteins. Manual alignment of elicitin-like sequences in OG5_133826 (the only elicitin-like family found in all ten oomycete secretomes) showed that 101 out of the 119 sequences contained the six necessary cysteine residues for proper folding and function (Jiang, Tyler, Whisson, et al. 2006; supplementary fig. S1, Supplementary Material online). Of the 18 sequences that were missing cysteine residues, six of them belong to *Ac. hypogyna* and four to *T*. *clavata*. Two sequences from *Ac. hypogyna*, one from *T*. *clavata*, and two from *S*. *parasitica* had the necessary sequence structure to be putative elicitin-like genes.

Further analysis of the *Ac. hypogyna* and *T*. *clavata* secretomes identified four other elicitin-like gene families, two from each species. These sequences contained all the necessary cysteine residues and were aligned with the other members of the gene family (supplementary fig. S2, Supplementary Material online). Additionally, several elicitin genes were present in transcriptome data (Misner et al. 2013), indicating this gene family is functional in a saprobic decomposer. This is the first report of elicitin-like genes occurring in a reportedly strictly saprotrophic oomycete. Although there are no confirmed reports of *T*. *clavata* acting as a pathogen, the possibility cannot be ruled out given taxonomic and identification uncertainties in the group that this species may capable of living as a pathogen. However, these data may suggest a more fundamental role for elicitin-like genes in lipid transport function, that can be functional in diverse ecologies including saprotrophic function (Blein et al. 2002; Jiang, Tyler, et al. 2006; Jiang, Tyler, Whisson, et al. 2006).

### Secretome Gene Gain, Duplication, and Loss

Of the 886 OrthoMCL groups (supplementary file S1, Supplementary Material online) that contain at least a single gene that putatively encodes a secreted protein, we were able to align and make phylogenetic trees for 695 groups. The NOTUNG analysis of 695 trees was used to identify gain, duplication, and loss events of all the putatively secreted orthologue families, throughout the oomycete phylogeny and therefore included both secreted (color values in fig. 1) and nonsecreted (black values in fig. 1) genes within each orthologue set. To identify evolutionary events within secretome genes only, all 695 trees analyzed by NOTUNG were manually analyzed to decipher evolutionary patterns. Secretome genes were classified into one of five categories and mapped on to figures 1 and 2. “Novel gain” was used for proteins that lack homologs in either the secretome or cytosolic proteome of taxa derived before the labeled node. “Gain from proteome” represents gene families that have cytosolic proteins in taxa derived prior to the labeled node, but are predicted by our pipeline as secreted, at the labeled node. “Duplications” are categorized by the duplication of secretome genes at the labeled node. “Loss to proteome” indicates proteins that are present in the genome, but no longer pass our secretome prediction pipeline. “Total loss” categorized proteins that could no longer be detected at, or after, a given node of the tree in either the secreted or cytosolic forms. This classification scheme was manually applied at each node of the tree (figs. 1 and 2).

Gains and losses were functionally compared by species (fig. 2), the largest changes occur in the KOG category of metabolism and those families involved in host–pathogen interactions (crinklers [CRN], RxLR, Elicitins, and necrosis-inducing peptides [NPP]). Each species has specific alterations of gene families involved in carbohydrate transport and metabolism (table 3)—a pattern consistent with a gain of carbohydrate transport and metabolism in the Peronosporalean clade, demonstrated by Richards et al. (2011). Oomycetes appear to be flexible in reconfiguring carbohydrate metabolism, which has undoubtedly led to successful lifestyles as osmotrophic feeders enabling host switching between plants and animals. However, most of the genes gained in any specific species did not have KOG classification hits (fig. 2).

**Table 3:** Distribution of Key CAZy Families by Species

Species	SAPpar	ACHhyp	THRcla	PHYcap	PHYinf	PHYram	PHYsoj	HYAara	PYTult	ALBcan	HYPcat
	Facultative	Facultative	Saprotroph	Hemibiotroph	Hemibiotroph	Hemibiotroph	Hemibiotroph	Obligate biotroph	Necrotroph	Obligate biotroph	Saprotroph
CBM family											
CBM1^AOS^	56	40	25	9	4	12	31	0	5	0	0
CBM13^A^^OS^	79	65	48	1	1	5	9	2	0	0	2
CBM18	1	2	9	0	0	0	0	0	0	0	0
CBM21	0	2	0	0	0	0	0	0	2	0	0
CBM24	0	1	0	1	0	1	0	0	0	0	0
CBM25	0	1	0	1	1	1	1	0	2	0	0
CBM32	1	1	0	1	1	0	2	0	0	0	0
CBM37^A^^OS^	0	1	0	1	0	0	1	0	0	0	0
CBM38	1	1	0	1	0	0	1	0	0	0	0
CBM40	0	1	0	1	0	1	0	0	1	0	0
CBM43	0	1	0	1	0	0	0	0	0	1	0
CBM47	1	1	0	0	1	0	2	0	0	0	0
CBM59	0	1	0	1	0	1	0	0	0	0	0
CBM63^A^^OS^	9	5	2	6	4	6	12	1	6	1	3
CBM9	1	1	0	1	1	1	0	0	0	0	0
CE family											
CE1^A^^OS^	0	1	0	3	2	5	14	1	1	0	4
CE10	2	2	0	4	0	8	18	0	1	0	3
CE12	0	1	0	1	0	1	4	1	0	0	0
CE13	0	4	0	8	4	5	10	0	4	0	0
CE14^A^^OS^	2	2	1	0	0	0	0	0	0	0	0
CE16	0	1	0	0	0	0	0	0	0	0	0
CE3	0	1	0	1	0	1	0	0	0	0	0
CE4	0	1	0	0	0	0	0	0	0	0	4
CE5^A^^OS^	0	1	0	6	4	4	29	1	0	0	0
CE7^A^^OS^	0	1	0	1	0	0	0	0	0	0	0
CE8	0	5	0	5	5	10	29	1	0	0	0
GH family											
GH1	0	1	0	3	1	4	6	0	0	0	1
GH10	0	2	0	5	2	5	5	1	0	0	0
GH105	0	1	0	1	0	1	1	0	0	0	0
GH11	1	1	2	0	0	0	0	0	0	0	0
GH114	0	1	0	1	1	0	1	0	0	0	0
GH12	0	5	0	13	5	5	22	2	0	0	0
GH13^A^^OS^	2	2	3	2	0	2	1	0	2	0	3
GH131	0	5	0	3	5	4	9	2	2	3	0
GH14^A^^OS^	2	1	2	0	0	0	0	0	0	0	2
GH15	0	2	0	0	0	0	0	0	2	0	0
GH16^A^^OS^	6	7	3	1	3	5	2	0	4	1	2
GH17^A^^OS^	19	19	16	10	9	19	37	4	9	0	3
GH18^AOS^^a^	11	8	6	2	1	2	3	1	1	0	3
GH19^A^^OS^	2	1	3	1	1	0	4	1	0	0	0
GH2	1	1	1	1	0	1	0	0	1	0	0
GH20	2	2	2	0	0	0	0	0	0	0	0
GH28^A^^OS^	2	1	1	18	12	13	40	1	3	3	0
GH3^A^^OS^	5	5	4	15	5	20	40	3	5	2	0
GH30^A^^OS^	0	1	0	11	9	8	22	3	4	0	1
GH31^A^^OS^	1	1	2	1	3	4	7	0	3	0	1
GH32	0	3	0	5	3	3	5	2	0	1	0
GH37^A^^OS^	1	1	0	0	1	1	2	1	0	0	1
GH38	2	2	0	0	1	1	2	0	1	0	1
GH43	0	1	1	6	1	7	17	1	0	0	0
GH45	0	1	0	1	0	0	1	0	0	0	0
GH46	0	1	0	0	0	0	0	0	0	0	1
GH47	2	4	1	1	0	0	2	0	0	0	0
GH5^A^^OS^	7	8	3	6	4	9	15	2	5	3	5
GH53	0	2	0	3	2	5	8	1	2	0	0
GH54	0	1	0	1	1	1	4	0	0	0	0
GH6^A^^OS^	6	5	5	4	5	8	8	2	1	0	2
GH62	1	1	0	0	0	0	0	0	0	0	0
GH63	1	1	1	0	0	0	0	0	0	0	0
GH7	0	1	0	4	2	1	7	1	1	1	1
GH72^A^^OS^	6	6	5	4	5	9	15	0	5	4	2
GH76	0	1	0	0	0	0	0	0	0	0	1
GH78	0	3	0	2	3	4	7	0	0	0	0
GH81^A^^OS^	2	2	1	4	6	8	10	1	4	2	0
GH85	0	1	0	0	0	0	1	0	0	0	0
GH89	2	3	2	2	0	1	1	0	1	0	0
GT family											
GT1	0	1	0	1	0	1	0	0	0	0	0
GT24	0	1	1	0	0	0	0	0	0	0	0
GT25	0	1	0	0	0	0	0	0	0	0	1
GT31	1	3	1	0	2	5	9	2	1	0	0
GT32	0	1	0	1	0	1	0	0	0	0	1
GT4	4	2	1	1	2	1	3	0	1	0	0
GT41	3	2	0	0	0	1	1	0	0	0	0
GT60	1	1	0	1	0	0	0	0	0	1	0
GT62	0	1	0	0	0	0	1	0	0	0	0
GT71	2	4	0	0	1	1	4	0	0	0	0
GT8	2	1	0	0	0	0	0	0	0	0	0
PL family											
PL1^A^^OS^^a^	3	1	2	17	11	14	42	3	11	0	0
PL12	1	1	0	0	0	0	0	0	0	0	0
PL14	0	1	0	0	0	0	0	0	0	0	1
PL3	0	18	0	19	18	14	49	6	13	0	0
PL4	0	3	0	4	3	5	4	0	2	0	0
PL7	0	1	0	0	0	0	0	0	0	0	0

Note.—Numbers represent protein copy number per species or *S. parasitica* (SAPpar), *Ac*. *hypogyna* (ACHhyp), *T*. *clavata* (THRcla), *Ph*. *capsici* (PHYcap), *Ph*. *infestans* (PHYinf), *Ph*. *ramorum* (PHYram), *Ph*. *sojae* (PHYsoj), *Hya*. *arabidopsis* (HYAara), *Py*. *ultimum* (PYTult), *Al*. *candida* (ALBcan), and *Hyp*. *catenoides* (HYPcat). ^AOS^ predicted in AOS.

^a^Predicted in HGT.

*Hyaloperonospora arabidopsis* had the largest number of secretome gene losses with 257, followed by *Ph. infestans* with 235 losses. With only 20 secretome genes gained, *Albugo candida* has the fewest secretome additions of all species studied. Within the Saprolegniales *T*. *clavata* had only 66 secretome gene gains. Notably, 49.9% (202 proteins) of the *T*. *clavata* secretome proteins placed in OrthoMCL groups were present in the AOS (supplementary file S1, Supplementary Material online). This is the highest percentage of any of the 11 species, suggesting little innovation in the secretome of this nonpathogenic decomposer. Similar to *T*. *clavata*, 35.7% of the *Ac. hypogyna* proteins and 31.4% of the *S*. *parasitica* proteins were found in AOS gene families. The patterns of gains and functionality of proteins between the two facultatively parasitic species, *Ac. hypogyna* and *S*. *parasitica* were comparable (fig. 2).

### CAZy Enzymes

The breakdown of carbohydrates in the extracellular environment is a key activity among osmotrophic feeders such as oomycetes (Richards and Talbot 2013). The CAZy database is a comprehensive archive of all known carbohydrate functioning enzymes including enzymes known to function in the breakdown of many cell wall structures such as pectin, hemicellulose, and cellulose found in plant cell walls and chitin and cellulose found in fungal and animal biological structures (O'Connell et al. 2012). The oomycete secretomes had numerous and diverse CAZy database profiles (Cantarel et al. 2009), which covered all five CAZy classes (table 3). CAZy enzymes are present in 30 AOS orthologue families covering all CAZy classes except glycosyltransferases with 33 unique CAZy families, 16 of which are glycoside hydrolases (GH; table 3). *Albugo candida* had the fewest CAZy enzymes (23), all present in AOS gene families. Two CAZy families GH18 and PL1 were shown to be derived from HGT events in our analysis (supplementary figs. S3 and S4, Supplementary Material online).

### Horizontal Gene Transfer

Phylogenetic analysis of the secretome gene families indicates multiple origins for both the AOS and putative secreted protein families acquired during the diversification of the oomycetes (fig. 1). Using a phylogenomic approach, we identified seven putative HGT events (supplementary fig. S3–S9, Supplementary Material online), six of which have not been previously reported. Three HGTs are from fungal lineages and four from bacterial lineages. Four of these transfers have been identified on the basis of robust phylogenetic relationships and three proposed based on restricted and discontinuous taxon distributions (supplementary table S1, Supplementary Material online). All of the putative HGT-derived gene families present in the AOS (three) are annotated as involved in pathogenicity and/or carbohydrate metabolism. Richards et al. (2011) previously suggested that pectate lyase (OG5_134795) was a putative transfer from fungi to the oomycetes. Our expanded genome sampling supports this conclusion (supplementary fig. S4, Supplementary Material online), indicating that pectate lyase was acquired deep within the radiation of the oomycetes at a similar position reported for a sugar transporter (Richards et al. 2011). The other two HGTs in the AOS were endoglucanase (OG5_137267) of bacterial origin, and carbohydrate-binding protein (OG5_136547), transferred from fungi (supplementary figs. S5 and S6, Supplementary Material online). The remaining four novel HGT events (supplementary figs. S3, S7–S9) were specific to Saprolegnialeans, with three bacterial and one fungal derived transfer (fig. 1).

## Discussion

### Ancestral Oomycete Secretome

Secreted proteins play a crucial biological role in the success of saprobes and parasites, as both types of organisms require nutrients and macromolecules from the extracellular environment. By identifying the secretomes of *Ac. hypogyna* and *T*. *clavata*, we are able to make direct comparisons to the secretomes of Peronosporalean oomycetes, and *Hyp*. *catenoides* to understand secretome evolution and determine the AOS. The variability of lifestyle, across two major lineages of oomycetes (Saprolegnialeans and the Peronosporaleans) and the putative sister group of the oomycetes (*Hyp*. *catenoides*) (Cavalier-Smith and Chao 2006), provides the most comprehensive examination of these crucial proteins to date. Functionally, the gene families of the AOS play putative roles primarily in carbohydrate and amino acid metabolism (fig. 2). This is consistent with the importance of the secretome of these microbes in acquiring extracellular nutrients (Richards and Talbot 2013). The carbohydrate metabolic functions are carried out by the numerous CAZy classified proteins, some of which are present in the AOS (table 3). Strikingly, a number of “effector” gene families that were previously believed to be derived features of the Peronosporaleans are also present in the AOS. These include CWDEs, protease inhibitors, Epi-like genes, oxidoreductases, and elicitin-like genes. Three of these gene families contain novel virulence genes identified by Raffaele et al. (2010), specifically a serine protease, pectate lyase, and carbonic anhydrase, which are located in the “plastic-secretome” of *Ph. infestans*.

The presence of effectors in the AOS supports the hypothesis presented by Beakes et al. (2012), that pathogenesis is the ancestral state and that the saprobic nature of *T*. *clavata* is a derived state. As mentioned earlier, the possibility exists that *T*. *clavata* is an unrecognized facultative pathogen; however, its reduced secretome could be interpreted as a sign it has lost or reduced many of the genes that encode functions to engage with host as a parasite. We were also able to identify mRNA products for 69% of the predicted secretome in *T*. *clavata* maintained in supplemented culture medium, versus expression of 42% of *Ac. hypogyna* secretome proteins in the same culture conditions. This may be the direct result of the lifestyle of each organism and the greater complexity of the *Ac. hypogyna* secretome. Functional analyses of these effector genes in *T*. *clavata* is necessary to identify the biological activity and localization of these proteins, which would shed more light onto the evolution of these virulent gene families. Additional sequencing of earlier diverging oomycete lineages will also help clarify the evolutionary innovation in infection mechanisms among this diverse and important lineage.

### Elicitin-Like Proteins in the Saprolegnialeans

After a thorough investigation of the *Phytophthora* EST database (Randall et al. 2005) and the genomes of *Ph. ramorum* and *Ph. sojae*, Jiang, Tyler, Whisson, (2006) determined that group-B and group-C elicitin and elicitin-like genes were not present outside of *Phytophthora* and *Pythium* species. However, in a recent study of the *S*. *parasitica* genome, 29 elicitin-like proteins were identified (Jiang et al. 2013). We have further identified 26 novel group-B elicitin-like genes in the *Ac*. *hypogyna* and *T*. *clavata* across four OrthoMCL gene families. Not all 26 have maintained the necessary cysteine residues for proper folding of the domain; 11 genes have the structure and sequence homology to be putative elicitin-like proteins (supplementary figs. S1 and S2, Supplementary Material online). Elicitin-like sequences are likely to play a role in lipid binding (Jiang, Tyler, Whisson, et al. 2006) and could explain their conservation in the Saprolegniaceae. Although all elicitin-like sequences have signal peptides, some elicitins have been localized to the extracellular surface where they function in sterol binding (Madoui et al. 2009). This extracellular membrane bound function is unlikely in the saprolegnialean elicitin-like genes as they lack identifiable transmembrane motifs that would be necessary for anchoring into the extracellular membrane, unless the protein binds to a second membrane bound protein.

### CAZy Secretome Evolution

It is clear that the AOS encompassed a large diversity of enzymes that functioned in degrading carbohydrates in the external environment a key requirement for osmotrophic function. CBM63, GH14, glycosyl transferase (GT) 24, and GT60 are exclusive to saprolegnialean taxa, whereas GH7, GH30 and the horizontally transferred GH10 (Richards et al. 2011) are specific to the Peronosporaleans. The AOS CAZy enzymes identified included proteins that are predicted to function in breaking down cellulose, hemicellulose, pectin, and chitin (table 3). Two GH families, GH18 and GH19, contain chitinases and are expanded with multiple copies in saprolegnialean taxa when compared with the Peronosporaleans. The process of chitin degradation is the primary reason that saprolegnialean taxa had more CAZy enzymes then members of the Peronosporaleans. Small amounts of chitin (<1% cell wall carbohydrates) have been reported in saprolegnian taxa (Guerriero et al. 2010). Additionally, many of the organisms fed upon by saprolegnian species contain chitin as an important, and sugar rich, biological structure.

The low number of CAZy enzymes in *Al*. *candida* is most likely related to its obligate biotrophic lifestyle. Reduction in the diversity of these enzyme families may be consistent with specialization and dependence on specific host-derived nutrient sources (Brunner et al. 2013). In comparison, the generalist *Phytophthora* spp. have been shown to encode an expanded CAZy repertoire (table 3; Ospina-Giraldo et al. 2010). As one example, *Ph. infestans* and *Pythium ultimum* have expanded polysaccharide lyase (PL) gene families compared with the saprolegnialean species. The secretome of the obligate biotroph, *Al*. *candida*, lacks all PL genes (table 3).

### Coupled Changes in Lifestyle and Secretomes

As evolutionary lineages undergo transitions between free-living and parasitic lifestyles the organisms face different challenges for nutrient acquisition and survival. Gene family expansion, via duplication, and contraction (gene loss) are two mechanisms by which an organism can adapt to these lifestyle changes and evolutionary pressures. Attempts have been made to track gene family evolution, broadly in the alveolates and stramenopiles (Martens et al. 2008), and more specifically in the oomycetes using stramenopile outgroups (Seidl et al. 2012; Adhikari et al. 2013). These analyses have demonstrated that lifestyle has an enormous impact on the protein family complement of an organism. However, analysis of oomycete taxa has been limited by both distant outgroups (diatoms) and nearly exclusive focus on the Peronosporalean taxa. The different lifestyles among the Saprolegnialean and Peronosporalean species are reflected in their respective secretome compositions at a finer scale than has been possible previously (figs. 1 and 2). The Saprolegnialean secretomes are dominated by proteins in small gene families that are related to carbohydrate metabolism and other metabolic functions indicative of a mostly saprobic lifestyle (fig. 2). In contrast, the Peronosporalean secretomes, although containing a diversity of proteins with putative metabolic functions, are characterized by an emergence of large pathogenic-functioning gene families (fig. 2). These large pathogenic gene families include CRN, NPP (likely acquired by HGT [Richards et al. 2011]), and RxLR effectors, all distinctive of hemibiotrophic and necrotrophic plant pathogens. Interestingly, these pathogenic genes do not appear to be derived from the AOS, but rather from secretome genes acquired later in the oomycete radiation (fig. 2). This functional shift from tightly regulated metabolic genes to large, highly divergent, pathogenic-trait associated gene families is characteristic of the change in evolutionary pressures associated with adopting a—virulent—pathogenic lifestyle (Morris et al. 2009; Raffaele and Kamoun 2012). Even more extreme secretome specialization is seen in the obligate biotroph *Al*. *candida* (fig. 2 and table 2). The *Al*. *candida* secretome features the highest number of outright gene family losses from the secretome, but also by far the highest number of redirection of cytosolic proteins to the secretome proteome (fig. 1).

Examinations of the secretome of Peronosporaleans have concentrated on expansions in particular gene families (Martens and Van de Peer 2010; Adhikari et al. 2013; Jiang et al. 2013). Perhaps surprisingly, the secretomes of *T*. *clavata* and *Ac. hypogyna* show very few expansions of AOS gene families outside of CAZy enzymes described below (supplementary file S1, Supplementary Material online). Therefore, it appears that the specializations associated with plant pathogenicity in the Peronosporaleans have been an enormous evolutionary driver of secretome specialization and expansion.

Additionally, these data suggest other mechanisms for lifestyle and host adaptation of the secretome, modification of existing proteins via the addition of signal peptides. All ten of the oomycetes in this study showed that some gene families were “gained” from the proteome (fig. 1). Although these “gains” are at the gene family level, it is intuitive to think some proteins would have had to acquire a signal peptide. Tonkin et al. (2008) demonstrated that signal peptides can originate via exon shuffling and random amino acid shuffling in *Plasmodium falciparum* and it is possible that similar evolutionary mechanisms are occurring in the oomycetes.

However, it should be noted that secretome gene prediction is predicated on the presence of the proper N-terminus in the protein sequence and therefore is open to errors if poor protein models are utilized. Incomplete protein models will, potentially, lack the complete N-terminal sequence and would therefore be lost by this approach. An investigation of *T*. *clavata* and *Ac. hypogyna* proteins revealed only 4.8% and 2.8% of these predicted proteins lacked a starting methionine, respectively. This figure is well within the boundaries of protein prediction from other oomycete genomes. After removing the approximately 20% of these proteins that have transmembrane domains from this subset and assuming no bias toward secretome proteins, the false negative rate is around 0.1% for *T*. *clavata* and *Ac. hypogyna*. Additionally, the absence of a start methionine does not exclude a protein from the secretome in this study, as all of the predicted secretomes include a low number of proteins lacking a start methionine.

### HGT’s Role in the Oomycete Secretome

Using a systematic phylogenetic analysis of the secretomes of *Ac. hypogyna* and *T*. *clavata*, we were able to assess the impact HGT has had on the origin and evolution of the oomycete secretome. We have identified six novel HGTs (supplementary table S1, Supplementary Material online) from bacterial or fungal lineages into oomycetes. Furthermore, we have identified three potential HGT events that produce complex evolutionary tree topologies (supplementary figs. S10–S12, Supplementary Material online). These genes could not be confidently assigned an origin. The number of HGTs described in this study significantly expands the number of secretome protein families that have HGT origins. Although the Richards et al. (2011) analyses included the entire predicted proteomes of four Peronosporalean oomycetes, it focused exclusively on HGT between fungi and oomycetes. Nonetheless, 17 of the 34 HGTs they identified included gene families that putatively encode secretome proteins. Four of the HGTs described here are specific to the Saprolegniaceae and imply that HGT between fungal and bacterial lineages played a role in genome evolution of the Saprolegniaceae. Fungal genes have played a key role in the secretome of plant pathogenic oomycetes, but with the exception of Belbahri et al. (2008), HGT events involving prokaryotes remain systematically underexplored in the Peronosporaleans. Transferred prokaryotic genes may be responsible for novel functions, as demonstrated in other eukaryotic lineages (Keeling and Palmer 2008; Marcet-Houben and Gabaldon 2010; Slot and Rokas 2011) and here for saprolegnialean taxa.

Jiang et. al. (2013) used a Hidden Markov Model approach to identify five putative horizontally transferred gene families in the *S*. *parasitica* genome. Using our phylogenetic pipeline, we reevaluated the five genes identified as HGTs in *S*. *parasitica* and found only one was identified in our analysis as a likely HGT with strong phylogenetic support (SPRG_08128; supplementary fig. S13, Supplementary Material online). Multiprotein alignments for a Laminin-like protein (SPRG_08424) and the Disintigren family (SPRG_14051) have low pairwise identity (6.7% and 12.1%, respectively) and low levels of identical sites (both 0.1%) to their hits in our phylogenetic database. These alignments result in phylogenetic tress with low bootstrap support values and their status as putative HGTs is not supported by our analysis. The CHAP protein family (SPRG_15528) has higher pairwise identity (averaging ∼34%) but the predicted protein in *S*. *parasitica* is 204 amino acids, whereas the most closely related sequences (and majority in the alignment) are three times that length. Although our pipeline does not identify SPRG_15528 as an HGT, we cannot exclude the possibility of HGT in the ancestry of this oomycete gene. The origin of the HylE gene (SPRG_04818) could not be determined, because the only identifiable homologs were from a handful of *Salmonella enterica* (*n* = 3) and *Escherichia coli* (*n* = 6) strains. Although the taxomic distribution of this gene family cannot exclude HGT, homology to proteins from only nine closely related prokaryotic strains is curious. There are no introns in this gene and it is encoded on a large contig with 276 other genes, suggesting the possibility of a recent transfer, but pairwise identity at the protein level with prokaryotic homologs is less than 22%. This is not be identified in our analysis as an HGT event.

Overall, the amount of HGT implicated in the oomycete secretome suggests that gene acquisition has played an important role in the evolution of the wider oomycete group. From their proposed ancestral forms as obligate pathogens of marine algae, oomycetes have diversified to be able to infect every major group of eukaryotes (Beakes et al. 2012). Combined with Richards et al. (2011), Belbahri et al. (2008), and Jiang et al. (2013) data reported here bring the number of secreted gene families that have xenic origins in oomycetes to 25 (26 if the CHAP gene family is included). These three studies demonstrate the important role that HGT has played in the evolution of pathogenic oomycetes. HGT has expanded the capacity for these microbes to process and take up sugar in the external environment, which is potentially consistent from their ancestral shift from photo/phagotrophs to osmotrophs. However, HGT is only part of the complex evolutionary patterns of the secretome.

## Conclusions

The evolution of the oomcyete secretome has been shaped by gene expansion by duplication, gene loss, and acquisition by HGT, much of which has occurred in response to changes in lifestyle and environment across the oomycetes. Based upon these analyses, the AOS was probably capable of supporting a free-living saprotroph, as there is little variance between gene families present in the AOS and the secretome of *T*. *clavata*. In contrast, the secretome of the obligate biotroph *Al*. *candida* contains numerous gene families not present in any of the other species investigated, supporting the notion that host/pathogen coevolution has had a profound effect on *Al*. *candida’s* genome content and putative secreted proteome. Furthermore, flexibility in carbohydrate metabolism, shown by the fact that over one-third of the AOS are CAZys, is another feature that has factored in the diversity of oomycetes and their host range. Secreted CAZy diversity has been linked to host/parasite coevolution (Brunner et al. 2013; Zheng et al. 2013), and the distinction between the Saprolegnialean and Peronosporalean profiles underscores their importance for oomycetes. The description of the secretome from plant infecting members of the Saprolegnialeans and animal infecting members of the Peronosporaleans will make it possible to separate evolutionary trends with each lineage from host-induced effects.

## Additional Data Files

The following additional data files are available with the online version of this paper. Additional data file 1 is a list of the genomes used in the phylogenetic analysis pipeline.

## Supplementary Material

Supplementary files S1 and S2 and figures S1–S13 are available at *Genome Biology and Evolution* online (http://www.gbe.oxfordjournals.org/).

Supplementary Data
