# Supplementary material for: The Secreted Proteins of Achlya hypogyna and Thraustotheca clavata Identify the Ancestral Oomycete Secretome and Reveal Gene Acquisitions by Horizontal Gene Transfer
Source: Genome Biol Evol. 2014 Dec 18;7(1):120–35. doi: 10.1093/gbe/evu276 (PMC4316629; doi:10.1093/gbe/evu276)
Supplement: Supplementary Data [file supp_7_1_120__index.html]

The Secreted Proteins of Achlya hypogyna and Thraustotheca clavata Identify the Ancestral Oomycete Secretome and Reveal Gene Acquisitions by Horizontal Gene Transfer — Supplementary Data 

# The Secreted Proteins of *Achlya hypogyna* and *Thraustotheca clavata* Identify the Ancestral Oomycete Secretome and Reveal Gene Acquisitions by Horizontal Gene Transfer

## Supplementary Data

files

**Files in this Data Supplement:**

- Supplementary Data - pdf file
- Supplementary Data - txt file
- Supplementary Data - xlsx file
- Supplementary Data - docx file
- Supplementary Data - docx file
- Supplementary Data - xlsx file
- Supplementary Data - txt file
