# Supplementary material for: The Secreted Proteins of Achlya hypogyna and Thraustotheca clavata Identify the Ancestral Oomycete Secretome and Reveal Gene Acquisitions by Horizontal Gene Transfer
Source: Genome Biol Evol. 2014 Dec 18;7(1):120–35. doi: 10.1093/gbe/evu276 (PMC4316629; doi:10.1093/gbe/evu276)
Supplement: Supplementary Data [file supp_evu276_evu276_Figures_GBE.pdf]

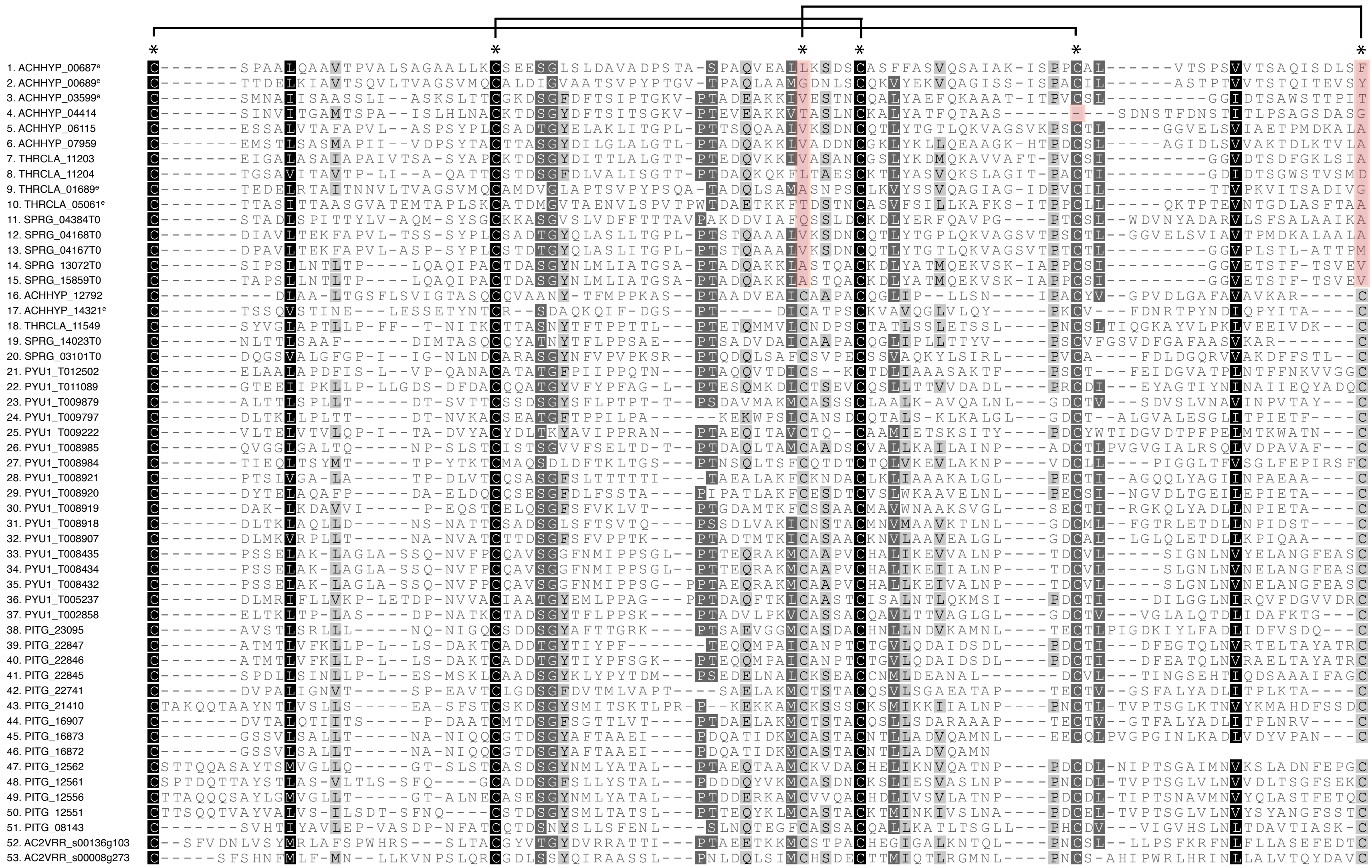
1. ACHHYP_00687[e]
2. ACHHYP_00689[e]
3. ACHHYP_03599[e]
4. ACHHYP_04414
5. ACHHYP_06115
6. ACHHYP_07959
7. THRCLA_11203
8. THRCLA_11204
9. THRCLA_01689[e]
10. THRCLA_05061[e]
11. SPRG_04384T0
12. SPRG_04168T0
13. SPRG_04167T0
14. SPRG_13072T0
15. SPRG_15859T0
16. ACHHYP_12792
17. ACHHYP_14321[e]
18. THRCLA_11549
19. SPRG_14023T0
20. SPRG_03101T0
21. PYU1_T012502
22. PYU1_T011089
23. PYU1_T009879
24. PYU1_T009797
25. PYU1_T009222
26. PYU1_T008985
27. PYU1_T008984
28. PYU1_T008921
29. PYU1_T008920
30. PYU1_T008919
31. PYU1_T008918
32. PYU1_T008907
33. PYU1_T008435
34. PYU1_T008434
35. PYU1_T008432
36. PYU1_T005237
37. PYU1_T002858
38. PITG_23095
39. PITG_22847
40. PITG_22846
41. PITG_22845
42. PITG_22741
43. PITG_21410
44. PITG_16907
45. PITG_16873
46. PITG_16872
47. PITG_12562
48. PITG_12561
49. PITG_12556
50. PITG_12551
51. PITG_08143
52. AC2VRR_s00136g103
53. AC2VRR_s00008g273

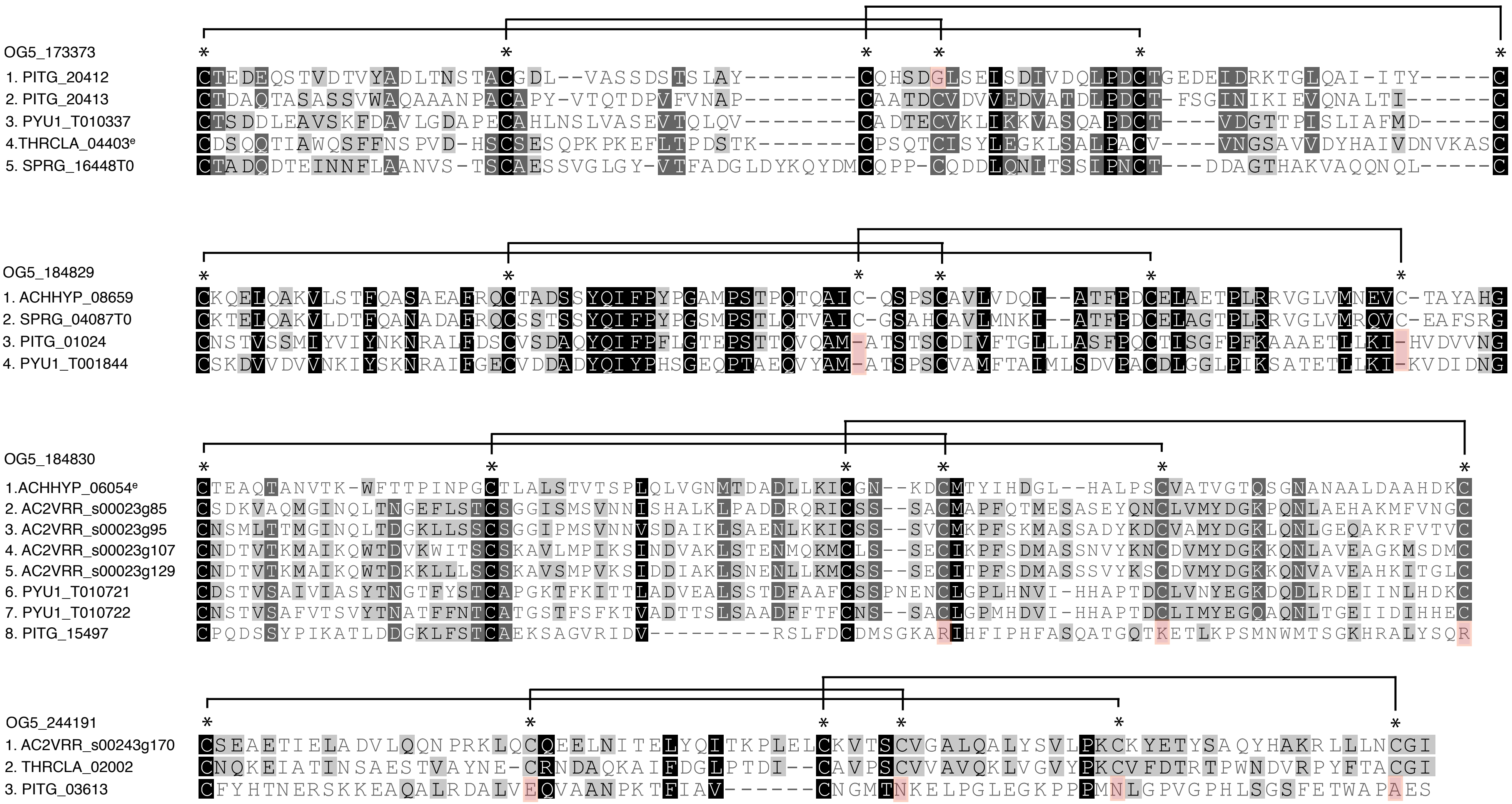
OG5_173373
1. PITG_20412 CTEDEQSTVDTVYADLTNSTACGDL--VASSDSTSLAY--------CQHSDGLSEISDIVDQLPDCTGEDEIDRKTGLQAI-ITY-----C
2. PITG_20413 CTDAQTASASSVWAQAAANPACAPY-VTQTDPVFVNAP---------CAATDCVDVVEDVATDLPDCT-FSGINIKIEVQNALTI------C
3. PYU1_T010337 CTSDDLEAVSKFDAVLGDAPECAHLNSLVASEVTQLQV--------CADTECVKLIKKVASQAPDCT----VDGTTPISLIAFMD-----C
4.THRCLA_04403[e] CDSQQTIAWQSFFNSPVD-HSCSESQPKPKEFLTPDSTK-------CPSQTCISYLEGKLSALPACV----VNGSAVVDYHAIVDNVKASC
5. SPRG_16448T0 CTADQDTEINNFLAANVS-TSCAESSVGLGY-VTFADGLDYKQYDMCQPP-CQDDLQNLTSSIPNCT---DDAGTHAKVAQQNQL-----C
OG5_184829
1. ACHHYP_08659 CKQELQAKVLSTFQASAEAFRQCTADSSYQIFPYPGAMPSTPQTQAIC-QSPSCAVLVDQI--ATFPDCELAETPLRRVGLVMNEVC-TAYAHG
2. SPRG_04087T0 CKTELQAKVLDTFQANADAFRQCSSTSSYQIFPYPGSMPSTLQTVAIC-GSAHCAVLMNKI--ATFPDCELAGTPLRRVGLVMRQVC-EAFSRG
3. PITG_01024 CNSTVSSMIYVIYNKNRALFDSCVSDAQYQIFPFLGTEPSTTQVQAM-ATSTSCDIVFTGLLLASFPQCTISGFPFKAAAETLLKI-HVDVVNG
4. PYU1_T001844 CSKDVVDVVNKIYSKNRAIFGECVDDADYQIYPHSGEQPTAEQVYAM-ATSPSCVAMFTAIMLSDVPACDLGGLPIKSATETLLKI-KVDIDNG
OG5_184830
1.ACHHYP_06054[e] CTEAQTANVTK-WFTTPINPGCTLALSTVTSPLQLVGNMTDADLLKICGN--KDCMTYIHDGL--HALPSCVATVGTQSGNANAALDAAHDKC
2. AC2VRR_s00023g85 CSDKVAQMGINQLTNGEFLSTCSGGISMSVNNISHALKLPADDRQRICSS--SACMAPFQTMESASEYQNCLVMYDGKPQNLAEHAKMFVNGC
3. AC2VRR_s00023g95 CNSMLTTMGINQLTDGKLLSSCSGGIPMSVNNVSDAIKLSAENLKKICSS--SVCMKPFSKMASSADYKDCVAMYDGKLQNLGEQAKRFVTVC
4. AC2VRR_s00023g107 CNDTVTKMAIKQWTDVKWITSCSKAVLMPIKSINDVAKLSTENMQKMCLS--SECIKPFSDMASSNVYKNCDVMYDGKKQNLAVEAGKMSDMC
5. AC2VRR_s00023g129 CNDTVTKMAIKQWTDKKLLLSCSKAVSMPVKSIDDIAKLSNENLLKMCSS--SECITPFSDMASSSVYKSCDVMYDGKKQNVAVEAHKITGLC
6. PYU1_T010721 CDSTVSAIVIASYTNGTFYSTCAPGKTFKITTLADVEALSSTDFAAFCSSPNENCLGPLHNVI-HHAPTDCLVNYEGKDQDLRDEIINLHDKC
7. PYU1_T010722 CNSTVSAFVTSVYTNATFFNTCATGSTFSFKTVADTTSLSAADFFTFCNS--SACLGPMHDVI-HHAPTDCLIMYEGQAQNLTGEIIDIHHEC
8. PITG_15497 CPQDSSYPIKATLDDGKLFSTCAEKSAGVRIDV---------RSLFDCDMSGKARIHFIPHFASQATGQTKETLKPSMNWMTSGKHRALYSQR
OG5_244191
1. AC2VRR_s00243g170 CSEAETIELADVLQQNPRKLQCQEELNITELYQITKPLELCKVTSCVGALQALYSVLPKCKYETYSAQYHAKRLLLNCGI
2. THRCLA_02002 CNQKEIATINSAESTVAYNE-CRNDAQKAIFDGLPTDI--CAVPSCVVAVQKLVGVYPKCVFDTRTPWNDVRPYFTACGI
3. PITG_03613 CFYHTNERSKKEAQALRDALVEQVAANPKTFIAV------CNGMTNKELPGLEGKPPMNLGPVGPHLSGSFETWAPAES

SI Figure 3

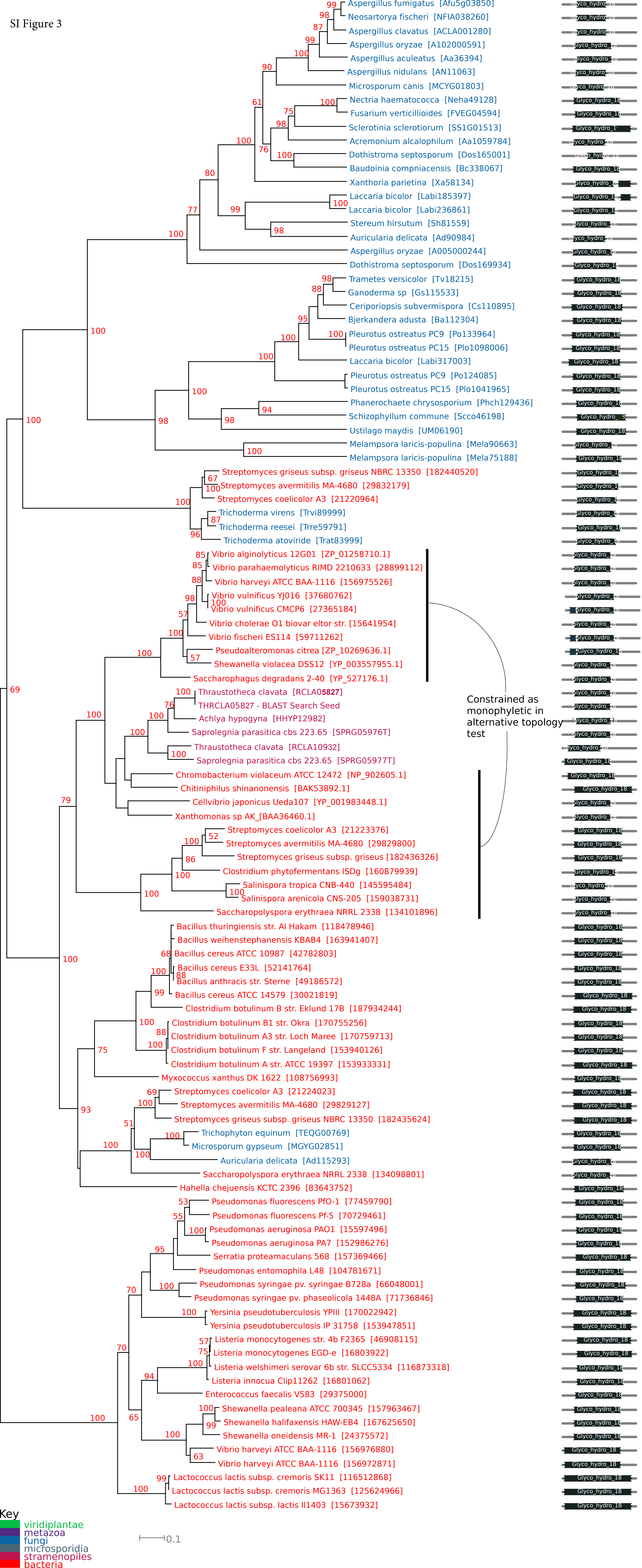

Aspergillus fumigatus [Afu5g03850]
Neosartorya fischeri [NFIA038260]
Aspergillus clavatus [ACLA001280]
Aspergillus oryzae [A102000591]
Aspergillus aculeatus [Aa36394]
Aspergillus nidulans [AN11063]
Microsporum canis [MCYG01803]
Nectria haematococca [Neha49128]
Fusarium verticillioides [FVEG04594]
Sclerotinia sclerotiorum [SS1G01513]
Acremonium alcalophilum [Aa1059784]
Dothistroma septosporum [Dos165001]
Baudoinia compniacensis [Bc338067]
Xanthoria parietina [Xa58134]
Laccaria bicolor [Labi185397]
Laccaria bicolor [Labi236861]
Stereum hirsutum [Sh81559]
Auricularia delicata [Ad90984]
Aspergillus oryzae [A005000244]
Dothistroma septosporum [Dos169934]
Trametes versicolor [Tv18215]
Ganoderma sp [Gs115533]
Ceriporiopsis subvermispora [Cs110895]
Bjerkandera adusta [Ba112304]
Pleurotus ostreatus PC9 [Po133964]
Pleurotus ostreatus PC15 [Plo1098006]
Laccaria bicolor [Labi317003]
Pleurotus ostreatus PC9 [Po124085]
Pleurotus ostreatus PC15 [Plo1041965]
Phanerochaete chrysosporium [Phch129436]
Schizophyllum commune [Scco46198]
Ustilago maydis [UM06190]
Melampsora laricis-populina [Mela90663]
Melampsora laricis-populina [Mela75188]
Streptomyces griseus subsp. griseus NBRC 13350 [182440520]
Streptomyces avermitilis MA-4680 [29832179]
Streptomyces coelicolor A3 [21220964]
Trichoderma virens [Trvi89999]
Trichoderma reesei [Trre59791]
Trichoderma atoviride [Trat83999]
Vibrio alginolyticus 12G01 [ZP_01258710.1]
Vibrio parahaemolyticus RIMD 2210633 [28899112]
Vibrio harveyi ATCC BAA-1116 [156975526]
Vibrio vulnificus YJ016 [37680762]
Vibrio vulnificus CMCP6 [27365184]
Vibrio cholerae O1 biovar eltor str. [15641954]
Vibrio fischeri ES114 [59711262]
Pseudoalteromonas citrea [ZP_10269636.1]
Shewanella violacea DSS12 [YP_003557955.1]
Saccharophagus degradans 2-40 [YP_527176.1]
Thraustotheca clavata [RCLA05827]
THRCLA05827 - BLAST Search Seed
Achlya hypogyna [HHYP12982]
Saprolegnia parasitica cbs 223.65 [SPRG05976T]
Thraustotheca clavata [RCLA10932]
Saprolegnia parasitica cbs 223.65 [SPRG05977T]
Chromobacterium violaceum ATCC 12472 [NP_902605.1]
Chitiniphilus shinanonensis [BAK53892.1]
Cellvibrio japonicus Ueda107 [YP_001983448.1]
Xanthomonas sp AK_[BAA36460.1]
Streptomyces coelicolor A3 [21223376]
Streptomyces avermitilis MA-4680 [29829800]
Streptomyces griseus subsp. griseus [182436326]
Clostridium phytofermentans ISDg [160879939]
Salinispora tropica CNB-440 [145595484]
Salinispora arenicola CNS-205 [159038731]
Saccharopolyspora erythraea NRRL 2338 [134101896]
Constrained as monophyletic in alternative topology test
Bacillus thuringiensis str. Al Hakam [118478946]
Bacillus weihenstephanensis KBAB4 [163941407]
Bacillus cereus ATCC 10987 [42782803]
Bacillus cereus E33L [52141764]
Bacillus anthracis str. Sterne [49186572]
Bacillus cereus ATCC 14579 [30021819]
Clostridium botulinum B str. Eklund 17B [187934244]
Clostridium botulinum B1 str. Okra [170755256]
Clostridium botulinum A3 str. Loch Maree [170759713]
Clostridium botulinum F str. Langeland [153940126]
Clostridium botulinum A str. ATCC 19397 [153933331]
Myxococcus xanthus DK 1622 [108756993]
Streptomyces coelicolor A3 [21224023]
Streptomyces avermitilis MA-4680 [29829127]
Streptomyces griseus subsp. griseus NBRC 13350 [182435624]
Trichophyton equinum [TEQG00769]
Microsporum gypseum [MGYG02851]
Auricularia delicata [Ad115293]
Saccharopolyspora erythraea NRRL 2338 [134098801]
Hahella chejuensis KCTC 2396 [83643752]
Pseudomonas fluorescens PfO-1 [77459790]
Pseudomonas fluorescens Pf-5 [70729461]
Pseudomonas aeruginosa PAO1 [15597496]
Pseudomonas aeruginosa PA7 [152986276]
Serratia proteamaculans 568 [157369466]
Pseudomonas entomophila L48 [104781671]
Pseudomonas syringae pv. syringae B728a [66048001]
Pseudomonas syringae pv. phaseolicola 1448A [71736846]
Yersinia pseudotuberculosis YPIII [170022942]
Yersinia pseudotuberculosis IP 31758 [153947851]
Listeria monocytogenes str. 4b F2365 [46908115]
Listeria monocytogenes EGD-e [16803922]
Listeria welshimeri serovar 6b str. SLCC5334 [116873318]
Listeria innocua Clip11262 [16801062]
Enterococcus faecalis V583 [29375000]
Shewanella pealeana ATCC 700345 [157963467]
Shewanella halifaxensis HAW-EB4 [167625650]
Shewanella oneidensis MR-1 [24375572]
Vibrio harveyi ATCC BAA-1116 [156976880]
Vibrio harveyi ATCC BAA-1116 [156972871]
Lactococcus lactis subsp. cremoris SK11 [116512868]
Lactococcus lactis subsp. cremoris MG1363 [125624966]
Lactococcus lactis subsp. lactis Il1403 [15673932]
Key
viridiplantae
metazoa
fungi
microsporidia
stramenopiles
bacteria
0.1

SI Figure 4

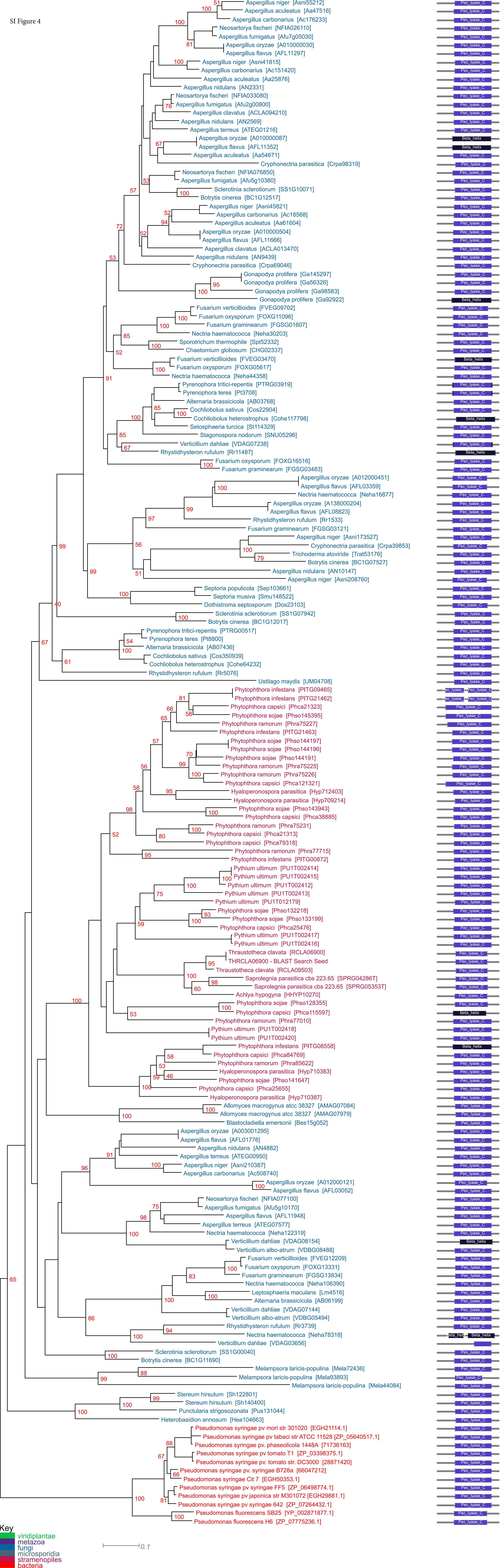
Aspergillus niger [Asni55212]
Aspergillus aculeatus [Aa47516]
Aspergillus carbonarius [Ac176233]
Neosartorya fischeri [NFIA026110]
Aspergillus fumigatus [Afu7g05030]
Aspergillus oryzae [A010000030]
Aspergillus flavus [AFL11297]
Aspergillus niger [Asni41815]
Aspergillus carbonarius [Ac151420]
Aspergillus aculeatus [Aa25876]
Aspergillus nidulans [AN2331]
Neosartorya fischeri [NFIA033080]
Aspergillus fumigatus [Afu2g00800]
Aspergillus clavatus [ACLA094210]
Aspergillus nidulans [AN2569]
Aspergillus terreus [ATEG01216]
Aspergillus oryzae [A010000087]
Aspergillus flavus [AFL11352]
Aspergillus aculeatus [Aa54671]
Cryphonectria parasitica [Crpa98319]
Neosartorya fischeri [NFIA076850]
Aspergillus fumigatus [Afu5g10380]
Sclerotinia sclerotiorum [SS1G10071]
Botrytis cinerea [BC1G12517]
Aspergillus niger [Asni45821]
Aspergillus carbonarius [Ac18568]
Aspergillus aculeatus [Aa61604]
Aspergillus oryzae [A010000504]
Aspergillus flavus [AFL11666]
Aspergillus clavatus [ACLA013470]
Aspergillus nidulans [AN9439]
Cryphonectria parasitica [Crpa69046]
Gonapodya prolifera [Ga145297]
Gonapodya prolifera [Ga56326]
Gonapodya prolifera [Ga98583]
Gonapodya prolifera [Ga92922]
Fusarium verticillioides [FVEG09702]
Fusarium oxysporum [FOXG11096]
Fusarium graminearum [FGSG01607]
Nectria haematococca [Neha30203]
Sporotrichum thermophile [Spt52332]
Chaetomium globosum [CHG02337]
Fusarium verticillioides [FVEG03470]
Fusarium oxysporum [FOXG05617]
Nectria haematococca [Neha44358]
Pyrenophora tritici-repentis [PTRG03919]
Pyrenophora teres [Pt3708]
Alternaria brassicicola [AB03768]
Cochliobolus sativus [Cos22904]
Cochliobolus heterostrophus [Cohe117798]
Setosphaeria turcica [St114329]
Stagonospora nodorum [SNU05296]
Verticillium dahliae [VDAG07238]
Rhystidhysteron rufulum [Rr11487]
Fusarium oxysporum [FOXG16516]
Fusarium graminearum [FGSG03483]
Aspergillus oryzae [A012000451]
Aspergillus flavus [AFL03359]
Nectria haematococca [Neha16877]
Aspergillus oryzae [A138000204]
Aspergillus flavus [AFL08823]
Rhystidhysteron rufulum [Rr1533]
Fusarium graminearum [FGSG03121]
Aspergillus niger [Asni173527]
Cryphonectria parasitica [Crpa39853]
Trichoderma atoviride [Trat53176]
Botrytis cinerea [BC1G07527]
Aspergillus nidulans [AN10147]
Aspergillus niger [Asni208760]
Septoria populicola [Sep103661]
Septoria musiva [Smu148522]
Dothistroma septosporum [Dos23103]
Sclerotinia sclerotiorum [SS1G07942]
Botrytis cinerea [BC1G12017]
Pyrenophora tritici-repentis [PTRG00517]
Pyrenophora teres [Pt8800]
Alternaria brassicicola [AB07436]
Cochliobolus sativus [Cos350939]
Cochliobolus heterostrophus [Cohe64232]
Rhystidhysteron rufulum [Rr5076]
Ustilago maydis [UM04708]
Phytophthora infestans [PITG09465]
Phytophthora infestans [PITG21462]
Phytophthora capsici [Phca21323]
Phytophthora sojae [Phso145395]
Phytophthora ramorum [Phra75227]
Phytophthora infestans [PITG21463]
Phytophthora sojae [Phso144197]
Phytophthora sojae [Phso144196]
Phytophthora sojae [Phso144191]
Phytophthora ramorum [Phra75225]
Phytophthora ramorum [Phra75226]
Phytophthora capsici [Phca121321]
Hyaloperonospora parasitica [Hyp712403]
Hyaloperonospora parasitica [Hyp709214]
Phytophthora sojae [Phso143943]
Phytophthora capsici [Phca38885]
Phytophthora ramorum [Phra75231]
Phytophthora capsici [Phca21313]
Phytophthora capsici [Phca79318]
Phytophthora ramorum [Phra77715]
Phytophthora infestans [PITG00872]
Pythium ultimum [PU1T002414]
Pythium ultimum [PU1T002415]
Pythium ultimum [PU1T002412]
Pythium ultimum [PU1T002413]
Pythium ultimum [PU1T012179]
Phytophthora sojae [Phso132218]
Phytophthora sojae [Phso133199]
Phytophthora capsici [Phca25476]
Pythium ultimum [PU1T002417]
Pythium ultimum [PU1T002416]
Thraustotheca clavata [RCLA06900]
THRCLA06900 - BLAST Search Seed
Thraustotheca clavata [RCLA09503]
Saprolegnia parasitica cbs 223.65 [SPRG04286T]
Saprolegnia parasitica cbs 223.65 [SPRG05353T]
Achlya hypogyna [HHYP10270]
Phytophthora sojae [Phso128355]
Phytophthora capsici [Phca115597]
Phytophthora ramorum [Phra77010]
Pythium ultimum [PU1T002418]
Pythium ultimum [PU1T002420]
Phytophthora infestans [PITG08558]
Phytophthora capsici [Phca84769]
Phytophthora ramorum [Phra85622]
Hyaloperonospora parasitica [Hyp710383]
Phytophthora sojae [Phso141647]
Phytophthora capsici [Phca25655]
Hyaloperonospora parasitica [Hyp710387]
Allomyces macrogynus atcc 38327 [AMAG07084]
Allomyces macrogynus atcc 38327 [AMAG07979]
Blastocladiella emersonii [Bes15g052]
Aspergillus oryzae [A003001295]
Aspergillus flavus [AFL01776]
Aspergillus nidulans [AN4882]
Aspergillus terreus [ATEG00950]
Aspergillus niger [Asni210387]
Aspergillus carbonarius [Ac508740]
Aspergillus oryzae [A012000121]
Aspergillus flavus [AFL03052]
Neosartorya fischeri [NFIA077100]
Aspergillus fumigatus [Afu5g10170]
Aspergillus flavus [AFL11948]
Aspergillus terreus [ATEG07577]
Nectria haematococca [Neha122319]
Verticillium dahliae [VDAG08154]
Verticillium albo-atrum [VDBG08488]
Fusarium verticillioides [FVEG12209]
Fusarium oxysporum [FOXG13331]
Fusarium graminearum [FGSG13834]
Nectria haematococca [Neha106390]
Leptosphaeria maculans [Lm4516]
Alternaria brassicicola [AB06199]
Verticillium dahliae [VDAG07144]
Verticillium albo-atrum [VDBG05494]
Rhystidhysteron rufulum [Rr3739]
Nectria haematococca [Neha78318]
Verticillium dahliae [VDAG03656]
Sclerotinia sclerotiorum [SS1G00040]
Botrytis cinerea [BC1G11690]
Melampsora laricis-populina [Mela72436]
Melampsora laricis-populina [Mela93893]
Melampsora laricis-populina [Mela44084]
Stereum hirsutum [Sh122801]
Stereum hirsutum [Sh140400]
Punctularia strigosozonata [Pus131044]
Heterobasidion annosum [Hea104663]
Pseudomonas syringae pv mori str 301020 [EGH21114.1]
Pseudomonas syringae pv tabaci str ATCC 11528 [ZP_05640517.1]
Pseudomonas syringae pv. phaseolicola 1448A [71736163]
Pseudomonas syringae pv tomato T1 [ZP_03398375.1]
Pseudomonas syringae pv. tomato str. DC3000 [28871420]
Pseudomonas syringae pv. syringae B728a [66047212]
Pseudomonas syringae Cit 7 [EGH50353.1]
Pseudomonas syringae pv syringae FF5 [ZP_06498774.1]
Pseudomonas syringae pv japonica str M301072 [EGH29881.1]
Pseudomonas syringae pv syringae 642 [ZP_07264432.1]
Pseudomonas fluorescens SB25 [YP_002871877.1]
Pseudomonas fluorescens H6 [ZP_07775236.1]
Pec_lyase_C
Beta_helix
0.1
Key
viridiplantae
metazoa
fungi
microsporidia
stramenopiles
bacteria

SI Figure 5

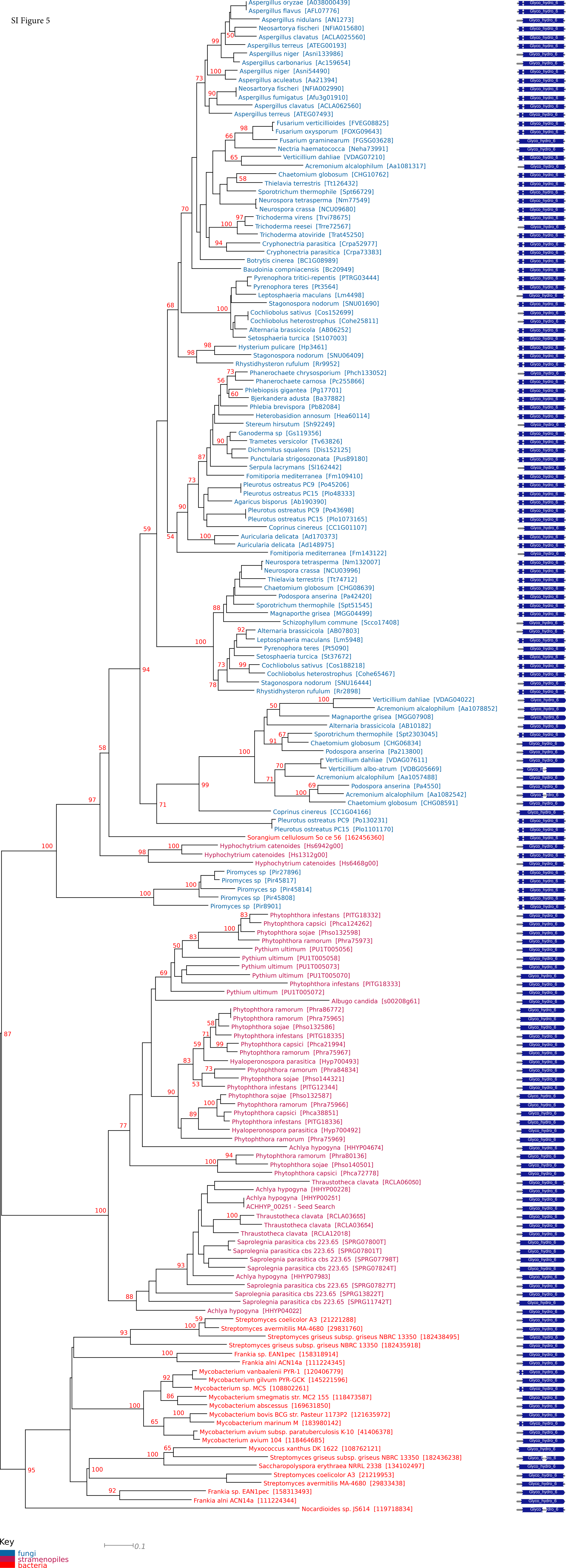
Aspergillus oryzae [AO38000439]
Aspergillus flavus [AFL07776]
Aspergillus nidulans [AN1273]
Neosartorya fischeri [NFIA015680]
Aspergillus clavatus [ACLA025560]
Aspergillus terreus [ATEG00193]
Aspergillus niger [Asni133986]
Aspergillus carbonarius [Ac159654]
Aspergillus niger [Asni54490]
Aspergillus aculeatus [Aa21394]
Neosartorya fischeri [NFIA002990]
Aspergillus fumigatus [Afu3g01910]
Aspergillus clavatus [ACLA062560]
Aspergillus terreus [ATEG07493]
Fusarium verticillioides [FVEG08825]
Fusarium oxysporum [FOXG09643]
Fusarium graminearum [FGSG03628]
Nectria haematococca [Neha73991]
Verticillium dahliae [VDAG07210]
Acremonium alcalophilum [Aa1081317]
Chaetomium globosum [CHG10762]
Thielavia terrestris [Tt126432]
Sporotrichum thermophile [Spt66729]
Neurospora tetrasperma [Nm77549]
Neurospora crassa [NCU09680]
Trichoderma virens [Trvi78675]
Trichoderma reesei [Trre72567]
Trichoderma atoviride [Trat45250]
Cryphonectria parasitica [Crpa52977]
Cryphonectria parasitica [Crpa73383]
Botrytis cinerea [BC1G08989]
Baudoinia compniacensis [Bc20949]
Pyrenophora tritici-repentis [PTRG03444]
Pyrenophora teres [Pt3564]
Leptosphaeria maculans [Lm4498]
Stagonospora nodorum [SNU01690]
Cochliobolus sativus [Cos152699]
Cochliobolus heterostrophus [Cohe25811]
Alternaria brassicicola [AB06252]
Setosphaeria turcica [St107003]
Hysterium pulicare [Hp3461]
Stagonospora nodorum [SNU06409]
Rhystidhysteron rufulum [Rr9952]
Phanerochaete chrysosporium [Phch133052]
Phanerochaete carnosa [Pc255866]
Phlebiopsis gigantea [Pg17701]
Bjerkandera adusta [Ba37882]
Phlebia brevispora [Pb82084]
Heterobasidion annosum [Hea60114]
Stereum hirsutum [Sh92249]
Ganoderma sp [Gs119356]
Trametes versicolor [Tv63826]
Dichomitus squalens [Dis152125]
Punctularia strigosozonata [Pus89180]
Serpula lacrymans [Sl162442]
Fomitiporia mediterranea [Fm109410]
Pleurotus ostreatus PC9 [Po45206]
Pleurotus ostreatus PC15 [Plo48333]
Agaricus bisporus [Ab190390]
Pleurotus ostreatus PC9 [Po43698]
Pleurotus ostreatus PC15 [Plo1073165]
Coprinus cinereus [CC1G01107]
Auricularia delicata [Ad170373]
Auricularia delicata [Ad148975]
Fomitiporia mediterranea [Fm143122]
Neurospora tetrasperma [Nm132007]
Neurospora crassa [NCU03996]
Thielavia terrestris [Tt74712]
Chaetomium globosum [CHG08639]
Podospora anserina [Pa42420]
Sporotrichum thermophile [Spt51545]
Magnaporthe grisea [MGG04499]
Schizophyllum commune [Scco17408]
Alternaria brassicicola [AB07803]
Leptosphaeria maculans [Lm5948]
Pyrenophora teres [Pt5090]
Setosphaeria turcica [St37672]
Cochliobolus sativus [Cos188218]
Cochliobolus heterostrophus [Cohe65467]
Stagonospora nodorum [SNU16444]
Rhystidhysteron rufulum [Rr2898]
Verticillium dahliae [VDAG04022]
Acremonium alcalophilum [Aa1078852]
Magnaporthe grisea [MGG07908]
Alternaria brassicicola [AB10182]
Sporotrichum thermophile [Spt2303045]
Chaetomium globosum [CHG06834]
Podospora anserina [Pa213800]
Verticillium dahliae [VDAG07611]
Verticillium albo-atrum [VDBG05669]
Acremonium alcalophilum [Aa1057488]
Podospora anserina [Pa4550]
Acremonium alcalophilum [Aa1082542]
Chaetomium globosum [CHG08591]
Coprinus cinereus [CC1G04166]
Pleurotus ostreatus PC9 [Po130231]
Pleurotus ostreatus PC15 [Plo1101170]
Sorangium cellulosum So ce 56 [162456360]
Hyphochytrium catenoides [Hs6942g00]
Hyphochytrium catenoides [Hs1312g00]
Hyphochytrium catenoides [Hs6468g00]
Piromyces sp [Pir27896]
Piromyces sp [Pir45817]
Piromyces sp [Pir45814]
Piromyces sp [Pir45808]
Piromyces sp [Pir8901]
Phytophthora infestans [PITG18332]
Phytophthora capsici [Phca124262]
Phytophthora sojae [Phso132598]
Phytophthora ramorum [Phra75973]
Pythium ultimum [PU1T005056]
Pythium ultimum [PU1T005058]
Pythium ultimum [PU1T005073]
Pythium ultimum [PU1T005070]
Phytophthora infestans [PITG18333]
Pythium ultimum [PU1T005072]
Albugo candida [s00208g61]
Phytophthora ramorum [Phra86772]
Phytophthora ramorum [Phra75965]
Phytophthora sojae [Phso132586]
Phytophthora infestans [PITG18335]
Phytophthora capsici [Phca21994]
Phytophthora ramorum [Phra75967]
Hyaloperonospora parasitica [Hyp700493]
Phytophthora ramorum [Phra84834]
Phytophthora sojae [Phso144321]
Phytophthora infestans [PITG12344]
Phytophthora sojae [Phso132587]
Phytophthora ramorum [Phra75966]
Phytophthora capsici [Phca38851]
Phytophthora infestans [PITG18336]
Hyaloperonospora parasitica [Hyp700492]
Phytophthora ramorum [Phra75969]
Achlya hypogyna [HHYP04674]
Phytophthora ramorum [Phra80136]
Phytophthora sojae [Phso140501]
Phytophthora capsici [Phca72778]
Thraustotheca clavata [RCLA06050]
Achlya hypogyna [HHYP00228]
Achlya hypogyna [HHYP00251]
ACHHYP_00251 - Seed Search
Thraustotheca clavata [RCLA03655]
Thraustotheca clavata [RCLA03654]
Thraustotheca clavata [RCLA12018]
Saprolegnia parasitica cbs 223.65 [SPRG07800T]
Saprolegnia parasitica cbs 223.65 [SPRG07801T]
Saprolegnia parasitica cbs 223.65 [SPRG07798T]
Saprolegnia parasitica cbs 223.65 [SPRG07824T]
Achlya hypogyna [HHYP07983]
Saprolegnia parasitica cbs 223.65 [SPRG07827T]
Saprolegnia parasitica cbs 223.65 [SPRG13822T]
Saprolegnia parasitica cbs 223.65 [SPRG11742T]
Achlya hypogyna [HHYP04022]
Streptomyces coelicolor A3 [21221288]
Streptomyces avermitilis MA-4680 [29831760]
Streptomyces griseus subsp. griseus NBRC 13350 [182438495]
Streptomyces griseus subsp. griseus NBRC 13350 [182435918]
Frankia sp. EAN1pec [158318914]
Frankia alni ACN14a [111224345]
Mycobacterium vanbaalenii PYR-1 [120406779]
Mycobacterium gilvum PYR-GCK [145221596]
Mycobacterium sp. MCS [108802261]
Mycobacterium smegmatis str. MC2 155 [118473587]
Mycobacterium abscessus [169631850]
Mycobacterium bovis BCG str. Pasteur 1173P2 [121635972]
Mycobacterium marinum M [183980142]
Mycobacterium avium subsp. paratuberculosis K-10 [41406378]
Mycobacterium avium 104 [118464685]
Myxococcus xanthus DK 1622 [108762121]
Streptomyces griseus subsp. griseus NBRC 13350 [182436238]
Saccharopolyspora erythraea NRRL 2338 [134102497]
Streptomyces coelicolor A3 [21219953]
Streptomyces avermitilis MA-4680 [29833438]
Frankia sp. EAN1pec [158313493]
Frankia alni ACN14a [111224344]
Nocardioides sp. JS614 [119718834]
Glyco_hydro_6
0.1
Key
fungi
stramenopiles
bacteria

SI Figure 6

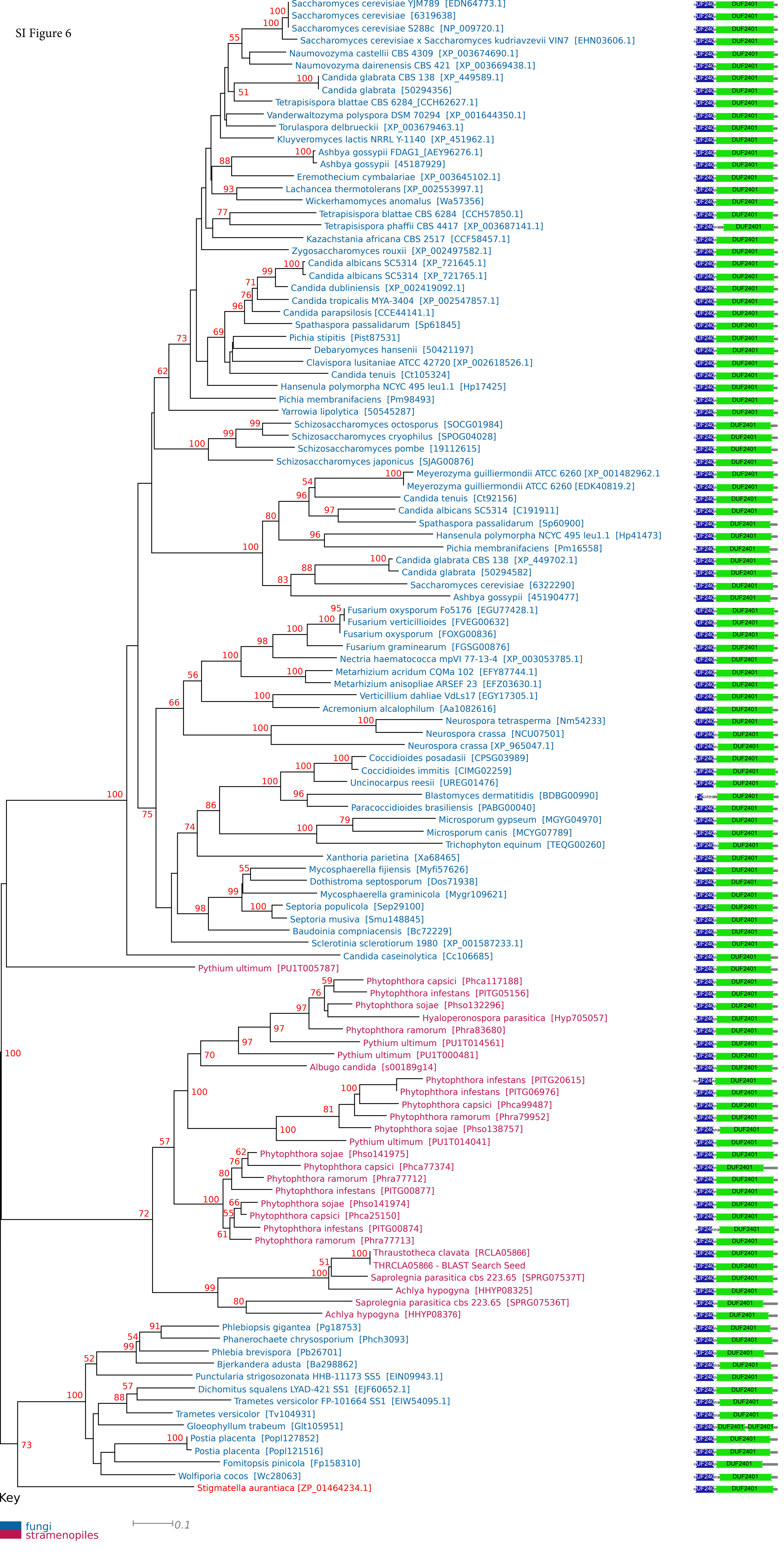

Saccharomyces cerevisiae YJM789 [EDN64773.1]
Saccharomyces cerevisiae [6319638]
Saccharomyces cerevisiae S288c [NP_009720.1]
Saccharomyces cerevisiae x Saccharomyces kudriavzevii VIN7 [EHN03606.1]
Naumovozyma castellii CBS 4309 [XP_003674690.1]
Naumovozyma dairenensis CBS 421 [XP_003669438.1]
Candida glabrata CBS 138 [XP_449589.1]
Candida glabrata [50294356]
Tetrapisispora blattae CBS 6284_[CCH62627.1]
Vanderwaltozyma polyspora DSM 70294 [XP_001644350.1]
Torulaspora delbrueckii [XP_003679463.1]
Kluyveromyces lactis NRRL Y-1140 [XP_451962.1]
Ashbya gossypii FDAG1_[AEY96276.1]
Ashbya gossypii [45187929]
Eremothecium cymbalariae [XP_003645102.1]
Lachancea thermotolerans [XP_002553997.1]
Wickerhamomyces anomalus [Wa57356]
Tetrapisispora blattae CBS 6284 [CCH57850.1]
Tetrapisispora phaffii CBS 4417 [XP_003687141.1]
Kazachstania africana CBS 2517 [CCF58457.1]
Zygosaccharomyces rouxii [XP_002497582.1]
Candida albicans SC5314 [XP_721645.1]
Candida albicans SC5314 [XP_721765.1]
Candida dubliniensis [XP_002419092.1]
Candida tropicalis MYA-3404 [XP_002547857.1]
Candida parapsilosis [CCE44141.1]
Spathaspora passalidarum [Sp61845]
Pichia stipitis [Pist87531]
Debaryomyces hansenii [50421197]
Clavispora lusitaniae ATCC 42720 [XP_002618526.1]
Candida tenuis [Ct105324]
Hansenula polymorpha NCYC 495 leu1.1 [Hp17425]
Pichia membranifaciens [Pm98493]
Yarrowia lipolytica [50545287]
Schizosaccharomyces octosporus [SOCG01984]
Schizosaccharomyces cryophilus [SPOG04028]
Schizosaccharomyces pombe [19112615]
Schizosaccharomyces japonicus [SJAG00876]
Meyerozyma guilliermondii ATCC 6260 [XP_001482962.1
Meyerozyma guilliermondii ATCC 6260 [EDK40819.2]
Candida tenuis [Ct92156]
Candida albicans SC5314 [C191911]
Spathaspora passalidarum [Sp60900]
Hansenula polymorpha NCYC 495 leu1.1 [Hp41473]
Pichia membranifaciens [Pm16558]
Candida glabrata CBS 138 [XP_449702.1]
Candida glabrata [50294582]
Saccharomyces cerevisiae [6322290]
Ashbya gossypii [45190477]
Fusarium oxysporum Fo5176 [EGU77428.1]
Fusarium verticillioides [FVEG00632]
Fusarium oxysporum [FOXG00836]
Fusarium graminearum [FGSG00876]
Nectria haematococca mpVI 77-13-4 [XP_003053785.1]
Metarhizium acridum CQMa 102 [EFY87744.1]
Metarhizium anisopliae ARSEF 23 [EFZ03630.1]
Verticillium dahliae VdLs17 [EGY17305.1]
Acremonium alcalophilum [Aa1082616]
Neurospora tetrasperma [Nm54233]
Neurospora crassa [NCU07501]
Neurospora crassa [XP_965047.1]
Coccidioides posadasii [CPSG03989]
Coccidioides immitis [CIMG02259]
Uncinocarpus reesii [UREG01476]
Blastomyces dermatitidis [BDBG00990]
Paracoccidioides brasiliensis [PABG00040]
Microsporum gypseum [MGYG04970]
Microsporum canis [MCYG07789]
Trichophyton equinum [TEQG00260]
Xanthoria parietina [Xa68465]
Mycosphaerella fijiensis [Myfi57626]
Dothistroma septosporum [Dos71938]
Mycosphaerella graminicola [Mygr109621]
Septoria populicola [Sep29100]
Septoria musiva [Smu148845]
Baudoinia compniacensis [Bc72229]
Sclerotinia sclerotiorum 1980 [XP_001587233.1]
Candida caseinolytica [Cc106685]
Pythium ultimum [PU1T005787]
Phytophthora capsici [Phca117188]
Phytophthora infestans [PITG05156]
Phytophthora sojae [Phso132296]
Hyaloperonospora parasitica [Hyp705057]
Phytophthora ramorum [Phra83680]
Pythium ultimum [PU1T014561]
Pythium ultimum [PU1T000481]
Albugo candida [s00189g14]
Phytophthora infestans [PITG20615]
Phytophthora infestans [PITG06976]
Phytophthora capsici [Phca99487]
Phytophthora ramorum [Phra79952]
Phytophthora sojae [Phso138757]
Pythium ultimum [PU1T014041]
Phytophthora sojae [Phso141975]
Phytophthora capsici [Phca77374]
Phytophthora ramorum [Phra77712]
Phytophthora infestans [PITG00877]
Phytophthora sojae [Phso141974]
Phytophthora capsici [Phca25150]
Phytophthora infestans [PITG00874]
Phytophthora ramorum [Phra77713]
Thraustotheca clavata [RCLA05866]
THRCLA05866 - BLAST Search Seed
Saprolegnia parasitica cbs 223.65 [SPRG07537T]
Achlya hypogyna [HHYP08325]
Saprolegnia parasitica cbs 223.65 [SPRG07536T]
Achlya hypogyna [HHYP08376]
Phlebiopsis gigantea [Pg18753]
Phanerochaete chrysosporium [Phch3093]
Phlebia brevispora [Pb26701]
Bjerkandera adusta [Ba298862]
Punctularia strigosozonata HHB-11173 SS5 [EIN09943.1]
Dichomitus squalens LYAD-421 SS1 [EJF60652.1]
Trametes versicolor FP-101664 SS1 [EIW54095.1]
Trametes versicolor [Tv104931]
Gloeophyllum trabeum [Glt105951]
Postia placenta [Popl127852]
Postia placenta [Popl121516]
Fomitopsis pinicola [Fp158310]
Wolfiporia cocos [Wc28063]
Stigmatella aurantiaca [ZP_01464234.1]
DUF2401
Key
fungi
stramenopiles
0.1

SI Figure 7

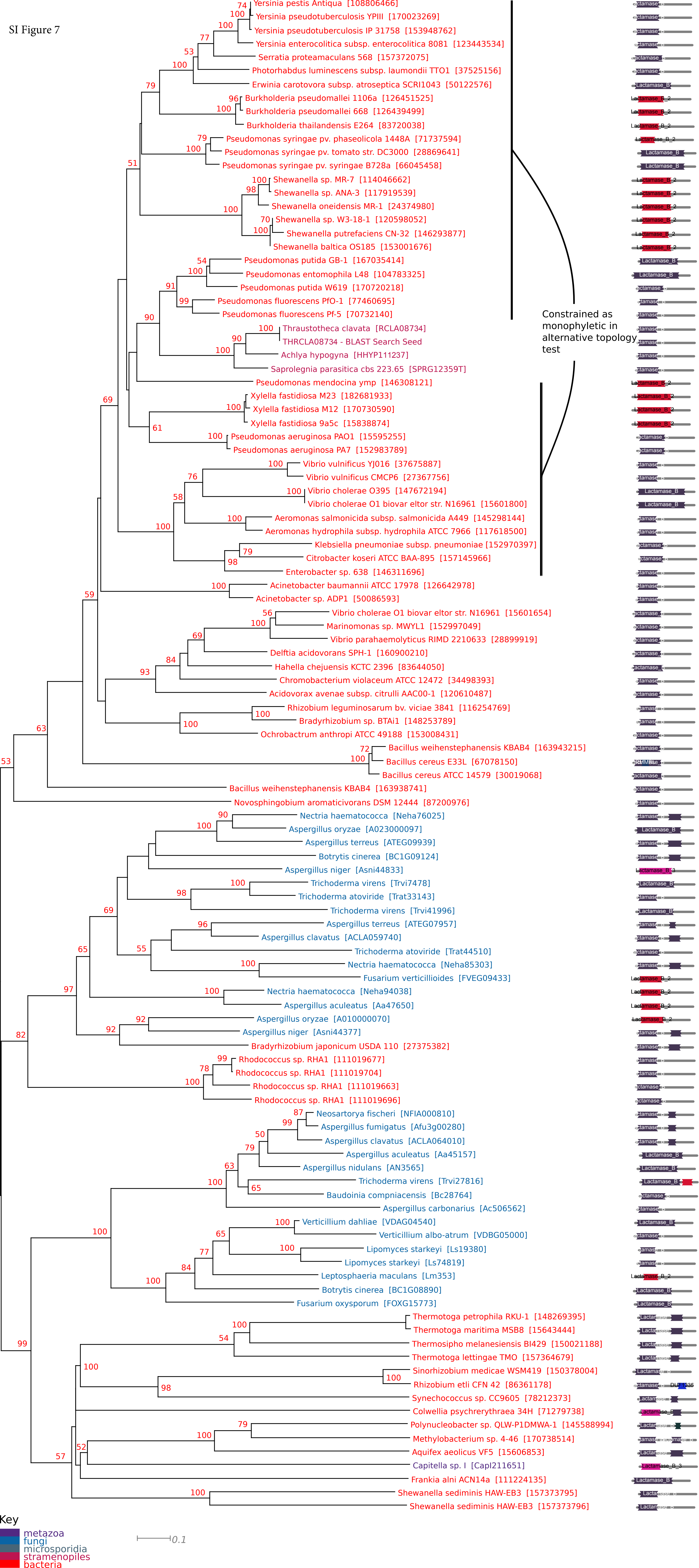
Yersinia pestis Antiqua [108806466]
Yersinia pseudotuberculosis YPIII [170023269]
Yersinia pseudotuberculosis IP 31758 [153948762]
Yersinia enterocolitica subsp. enterocolitica 8081 [123443534]
Serratia proteamaculans 568 [157372075]
Photorhabdus luminescens subsp. laumondii TTO1 [37525156]
Erwinia carotovora subsp. atroseptica SCRI1043 [50122576]
Burkholderia pseudomallei 1106a [126451525]
Burkholderia pseudomallei 668 [126439499]
Burkholderia thailandensis E264 [83720038]
Pseudomonas syringae pv. phaseolicola 1448A [71737594]
Pseudomonas syringae pv. tomato str. DC3000 [28869641]
Pseudomonas syringae pv. syringae B728a [66045458]
Shewanella sp. MR-7 [114046662]
Shewanella sp. ANA-3 [117919539]
Shewanella oneidensis MR-1 [24374980]
Shewanella sp. W3-18-1 [120598052]
Shewanella putrefaciens CN-32 [146293877]
Shewanella baltica OS185 [153001676]
Pseudomonas putida GB-1 [167035414]
Pseudomonas entomophila L48 [104783325]
Pseudomonas putida W619 [170720218]
Pseudomonas fluorescens PfO-1 [77460695]
Pseudomonas fluorescens Pf-5 [70732140]
Thraustotheca clavata [RCLA08734]
THRCLA08734 - BLAST Search Seed
Achlya hypogyna [HHYP111237]
Saprolegnia parasitica cbs 223.65 [SPRG12359T]
Pseudomonas mendocina ymp [146308121]
Xylella fastidiosa M23 [182681933]
Xylella fastidiosa M12 [170730590]
Xylella fastidiosa 9a5c [15838874]
Pseudomonas aeruginosa PAO1 [15595255]
Pseudomonas aeruginosa PA7 [152983789]
Vibrio vulnificus YJ016 [37675887]
Vibrio vulnificus CMCP6 [27367756]
Vibrio cholerae O395 [147672194]
Vibrio cholerae O1 biovar eltor str. N16961 [15601800]
Aeromonas salmonicida subsp. salmonicida A449 [145298144]
Aeromonas hydrophila subsp. hydrophila ATCC 7966 [117618500]
Klebsiella pneumoniae subsp. pneumoniae [152970397]
Citrobacter koseri ATCC BAA-895 [157145966]
Enterobacter sp. 638 [146311696]
Acinetobacter baumannii ATCC 17978 [126642978]
Acinetobacter sp. ADP1 [50086593]
Vibrio cholerae O1 biovar eltor str. N16961 [15601654]
Marinomonas sp. MWYL1 [152997049]
Vibrio parahaemolyticus RIMD 2210633 [28899919]
Delftia acidovorans SPH-1 [160900210]
Hahella chejuensis KCTC 2396 [83644050]
Chromobacterium violaceum ATCC 12472 [34498393]
Acidovorax avenae subsp. citrulli AAC00-1 [120610487]
Rhizobium leguminosarum bv. viciae 3841 [116254769]
Bradyrhizobium sp. BTAi1 [148253789]
Ochrobactrum anthropi ATCC 49188 [153008431]
Bacillus weihenstephanensis KBAB4 [163943215]
Bacillus cereus E33L [67078150]
Bacillus cereus ATCC 14579 [30019068]
Bacillus weihenstephanensis KBAB4 [163938741]
Novosphingobium aromaticivorans DSM 12444 [87200976]
Nectria haematococca [Neha76025]
Aspergillus oryzae [A023000097]
Aspergillus terreus [ATEG09939]
Botrytis cinerea [BC1G09124]
Aspergillus niger [Asni44833]
Trichoderma virens [Trvi7478]
Trichoderma atoviride [Trat33143]
Trichoderma virens [Trvi41996]
Aspergillus terreus [ATEG07957]
Aspergillus clavatus [ACLA059740]
Trichoderma atoviride [Trat44510]
Nectria haematococca [Neha85303]
Fusarium verticillioides [FVEG09433]
Nectria haematococca [Neha94038]
Aspergillus aculeatus [Aa47650]
Aspergillus oryzae [A010000070]
Aspergillus niger [Asni44377]
Bradyrhizobium japonicum USDA 110 [27375382]
Rhodococcus sp. RHA1 [111019677]
Rhodococcus sp. RHA1 [111019704]
Rhodococcus sp. RHA1 [111019663]
Rhodococcus sp. RHA1 [111019696]
Neosartorya fischeri [NFIA000810]
Aspergillus fumigatus [Afu3g00280]
Aspergillus clavatus [ACLA064010]
Aspergillus aculeatus [Aa45157]
Aspergillus nidulans [AN3565]
Trichoderma virens [Trvi27816]
Baudoinia compniacensis [Bc28764]
Aspergillus carbonarius [Ac506562]
Verticillium dahliae [VDAG04540]
Verticillium albo-atrum [VDBG05000]
Lipomyces starkeyi [Ls19380]
Lipomyces starkeyi [Ls74819]
Leptosphaeria maculans [Lm353]
Botrytis cinerea [BC1G08890]
Fusarium oxysporum [FOXG15773]
Thermotoga petrophila RKU-1 [148269395]
Thermotoga maritima MSB8 [15643444]
Thermosipho melanesiensis BI429 [150021188]
Thermotoga lettingae TMO [157364679]
Sinorhizobium medicae WSM419 [150378004]
Rhizobium etli CFN 42 [86361178]
Synechococcus sp. CC9605 [78212373]
Colwellia psychrerythraea 34H [71279738]
Polynucleobacter sp. QLW-P1DMWA-1 [145588994]
Methylobacterium sp. 4-46 [170738514]
Aquifex aeolicus VF5 [15606853]
Capitella sp. I [CapI211651]
Frankia alni ACN14a [111224135]
Shewanella sediminis HAW-EB3 [157373795]
Shewanella sediminis HAW-EB3 [157373796]
Constrained as monophyletic in alternative topology test
Key
metazoa
fungi
microsporidia
stramenopiles
bacteria
0.1

SI Figure 8

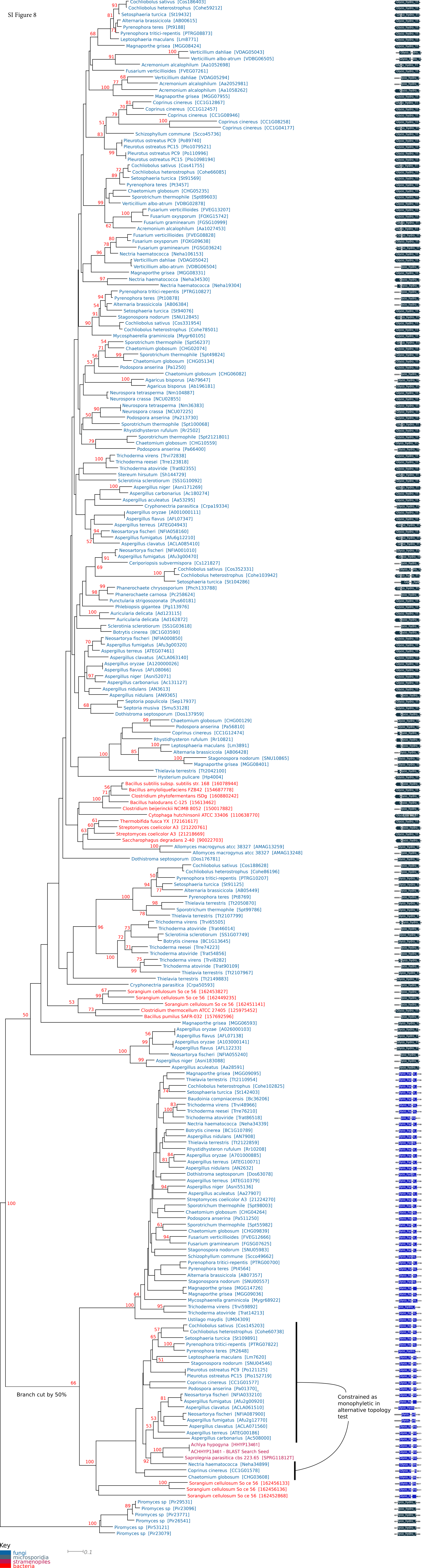

Cochliobolus sativus [Cos186403]
Cochliobolus heterostrophus [Cohe59212]
Setosphaeria turcica [St19432]
Alternaria brassicicola [AB00615]
Pyrenophora teres [Pt9188]
Pyrenophora tritici-repentis [PTRG08873]
Leptosphaeria maculans [Lm8771]
Magnaporthe grisea [MGG08424]
Verticillium dahliae [VDAG05043]
Verticillium albo-atrum [VDBG06505]
Acremonium alcalophilum [Aa1052698]
Fusarium verticillioides [FVEG07261]
Verticillium dahliae [VDAG05294]
Acremonium alcalophilum [Aa2052981]
Acremonium alcalophilum [Aa1058262]
Magnaporthe grisea [MGG07955]
Coprinus cinereus [CC1G12867]
Coprinus cinereus [CC1G12457]
Coprinus cinereus [CC1G08946]
Coprinus cinereus [CC1G08258]
Coprinus cinereus [CC1G04177]
Schizophyllum commune [Scco45736]
Pleurotus ostreatus PC9 [Po89740]
Pleurotus ostreatus PC15 [Plo1079521]
Pleurotus ostreatus PC9 [Po110996]
Pleurotus ostreatus PC15 [Plo1098194]
Cochliobolus sativus [Cos41755]
Cochliobolus heterostrophus [Cohe66085]
Setosphaeria turcica [St91569]
Pyrenophora teres [Pt3457]
Chaetomium globosum [CHG05235]
Sporotrichum thermophile [Spt89603]
Verticillium albo-atrum [VDBG02878]
Fusarium verticillioides [FVEG13207]
Fusarium oxysporum [FOXG15742]
Fusarium graminearum [FGSG10999]
Acremonium alcalophilum [Aa1027453]
Fusarium verticillioides [FVEG08828]
Fusarium oxysporum [FOXG09638]
Fusarium graminearum [FGSG03624]
Nectria haematococca [Neha106153]
Verticillium dahliae [VDAG05042]
Verticillium albo-atrum [VDBG06504]
Magnaporthe grisea [MGG08331]
Nectria haematococca [Neha34530]
Nectria haematococca [Neha19304]
Pyrenophora tritici-repentis [PTRG10827]
Pyrenophora teres [Pt10878]
Alternaria brassicicola [AB06384]
Setosphaeria turcica [St94076]
Stagonospora nodorum [SNU12845]
Cochliobolus sativus [Cos331954]
Cochliobolus heterostrophus [Cohe78501]
Mycosphaerella graminicola [Mygr60105]
Sporotrichum thermophile [Spt56237]
Chaetomium globosum [CHG02074]
Sporotrichum thermophile [Spt49824]
Chaetomium globosum [CHG05134]
Podospora anserina [Pa1250]
Chaetomium globosum [CHG06082]
Agaricus bisporus [Ab79647]
Agaricus bisporus [Ab196181]
Neurospora tetrasperma [Nm104887]
Neurospora crassa [NCU02855]
Neurospora tetrasperma [Nm36383]
Neurospora crassa [NCU07225]
Podospora anserina [Pa213730]
Sporotrichum thermophile [Spt100068]
Rhytidhysteron rufulum [Rr2502]
Sporotrichum thermophile [Spt2121801]
Chaetomium globosum [CHG10559]
Podospora anserina [Pa66400]
Trichoderma virens [Trvi72838]
Trichoderma reesei [Trre123818]
Trichoderma atoviride [Trat82355]
Stereum hirsutum [Sh144729]
Sclerotinia sclerotiorum [SS1G10092]
Aspergillus niger [Asni171269]
Aspergillus carbonarius [Ac180274]
Aspergillus aculeatus [Aa53295]
Cryphonectria parasitica [Crpa19334]
Aspergillus oryzae [A001000111]
Aspergillus flavus [AFL07347]
Aspergillus terreus [ATEG04943]
Neosartorya fischeri [NFIA058160]
Aspergillus fumigatus [Afu6g12210]
Aspergillus clavatus [ACLA085410]
Neosartorya fischeri [NFIA001010]
Aspergillus fumigatus [Afu3g00470]
Ceriporiopsis subvermispora [Cs121827]
Cochliobolus sativus [Cos352331]
Cochliobolus heterostrophus [Cohe103942]
Setosphaeria turcica [St104286]
Phanerochaete chrysosporium [Phch133788]
Phanerochaete carnosa [Pc258624]
Punctularia strigosozonata [Pus60181]
Phlebiopsis gigantea [Pg113976]
Auricularia delicata [Ad123115]
Auricularia delicata [Ad162872]
Sclerotinia sclerotiorum [SS1G03618]
Botrytis cinerea [BC1G03590]
Neosartorya fischeri [NFIA000850]
Aspergillus fumigatus [Afu3g00320]
Aspergillus terreus [ATEG07461]
Aspergillus clavatus [ACLA063140]
Aspergillus oryzae [A120000026]
Aspergillus flavus [AFL08066]
Aspergillus niger [Asni52071]
Aspergillus carbonarius [Ac131127]
Aspergillus nidulans [AN3613]
Aspergillus nidulans [AN9365]
Septoria populicola [Sep17937]
Septoria musiva [Smu53128]
Dothistroma septosporum [Dos137959]
Chaetomium globosum [CHG00129]
Podospora anserina [Pa56810]
Coprinus cinereus [CC1G12474]
Rhytidhysteron rufulum [Rr10821]
Leptosphaeria maculans [Lm3891]
Alternaria brassicicola [AB06428]
Stagonospora nodorum [SNU10865]
Magnaporthe grisea [MGG08401]
Thielavia terrestris [Tt2042100]
Hysterium pulicare [Hp4004]
Bacillus subtilis subsp. subtilis str. 168 [16078944]
Bacillus amyloliquefaciens FZB42 [154687778]
Clostridium phytofermentans ISDg [160880242]
Bacillus halodurans C-125 [15613462]
Clostridium beijerinckii NCIMB 8052 [150017882]
Cytophaga hutchinsonii ATCC 33406 [110638770]
Thermobifida fusca YX [72161617]
Streptomyces coelicolor A3 [21220761]
Streptomyces coelicolor A3 [21218669]
Saccharophagus degradans 2-40 [90022703]
Allomyces macrogynus atcc 38327 [AMAG13259]
Allomyces macrogynus atcc 38327 [AMAG13248]
Dothistroma septosporum [Dos176781]
Cochliobolus sativus [Cos188628]
Cochliobolus heterostrophus [Cohe86196]
Pyrenophora tritici-repentis [PTRG10207]
Setosphaeria turcica [St91125]
Alternaria brassicicola [AB05449]
Pyrenophora teres [Pt8769]
Thielavia terrestris [Tt2050870]
Sporotrichum thermophile [Spt99786]
Thielavia terrestris [Tt2107799]
Trichoderma virens [Trvi65505]
Trichoderma atoviride [Trat46014]
Sclerotinia sclerotiorum [SS1G07749]
Botrytis cinerea [BC1G13645]
Trichoderma reesei [Trre74223]
Trichoderma atoviride [Trat54856]
Trichoderma virens [Trvi8282]
Trichoderma atoviride [Trat90109]
Thielavia terrestris [Tt2107967]
Thielavia terrestris [Tt2149883]
Cryphonectria parasitica [Crpa50593]
Sorangium cellulosum So ce 56 [162453827]
Sorangium cellulosum So ce 56 [162449235]
Sorangium cellulosum So ce 56 [162451141]
Clostridium thermocellum ATCC 27405 [125975452]
Bacillus pumilus SAFR-032 [157692596]
Magnaporthe grisea [MGG06593]
Aspergillus oryzae [A026000103]
Aspergillus flavus [AFL07138]
Aspergillus oryzae [A103000141]
Aspergillus flavus [AFL12233]
Neosartorya fischeri [NFIA055240]
Aspergillus niger [Asni183088]
Aspergillus aculeatus [Aa28591]
Magnaporthe grisea [MGG09095]
Thielavia terrestris [Tt2110954]
Cochliobolus heterostrophus [Cohe102825]
Setosphaeria turcica [St142403]
Baudoinia compniacensis [Bc36206]
Trichoderma virens [Trvi48966]
Trichoderma reesei [Trre76210]
Trichoderma atoviride [Trat86518]
Nectria haematococca [Neha34339]
Botrytis cinerea [BC1G10789]
Aspergillus nidulans [AN7908]
Thielavia terrestris [Tt2122859]
Rhytidhysteron rufulum [Rr10208]
Aspergillus oryzae [A701000885]
Aspergillus terreus [ATEG10071]
Aspergillus nidulans [AN2632]
Dothistroma septosporum [Dos63078]
Aspergillus terreus [ATEG10379]
Aspergillus niger [Asni55136]
Aspergillus aculeatus [Aa27907]
Streptomyces coelicolor A3 [21224270]
Sporotrichum thermophile [Spt98003]
Chaetomium globosum [CHG04264]
Podospora anserina [Pa511250]
Sporotrichum thermophile [Spt55982]
Chaetomium globosum [CHG09839]
Fusarium verticillioides [FVEG12666]
Fusarium graminearum [FGSG07625]
Stagonospora nodorum [SNU05983]
Schizophyllum commune [Scco49662]
Pyrenophora tritici-repentis [PTRG00700]
Pyrenophora teres [Pt4564]
Alternaria brassicicola [AB07357]
Stagonospora nodorum [SNU00557]
Magnaporthe grisea [MGG14726]
Magnaporthe grisea [MGG09036]
Mycosphaerella graminicola [Mygr68922]
Trichoderma virens [Trvi59892]
Trichoderma atoviride [Trat14213]
Ustilago maydis [UM04309]
Cochliobolus sativus [Cos145203]
Cochliobolus heterostrophus [Cohe60738]
Setosphaeria turcica [St109891]
Pyrenophora tritici-repentis [PTRG07822]
Pyrenophora teres [Pt2648]
Leptosphaeria maculans [Lm7620]
Stagonospora nodorum [SNU04546]
Pleurotus ostreatus PC9 [Po121125]
Pleurotus ostreatus PC15 [Plo152719]
Coprinus cinereus [CC1G01577]
Podospora anserina [Pa01370]
Neosartorya fischeri [NFIA033210]
Aspergillus fumigatus [Afu2g00920]
Aspergillus clavatus [ACLA061510]
Neosartorya fischeri [NFIA087900]
Aspergillus fumigatus [Afu2g12770]
Aspergillus clavatus [ACLA071560]
Aspergillus terreus [ATEG00186]
Aspergillus carbonarius [Ac508000]
Achlya hypogyna [HHYP13461]
ACHHYP13461 - BLAST Search Seed
Saprolegnia parasitica cbs 223.65 [SPRG11812T]
Nectria haematococca [Neha34899]
Coprinus cinereus [CC1G01578]
Chaetomium globosum [CHG03608]
Sorangium cellulosum So ce 56 [162456133]
Sorangium cellulosum So ce 56 [162456136]
Sorangium cellulosum So ce 56 [162452868]
Piromyces sp [Pir29531]
Piromyces sp [Pir23096]
Piromyces sp [Pir23771]
Piromyces sp [Pir26541]
Piromyces sp [Pir53121]
Piromyces sp [Pir23079]
Branch cut by 50%
Constrained as monophyletic in alternative topology test
Key
fungi
microsporidia
stramenopiles
bacteria
0.1

SI Figure 9

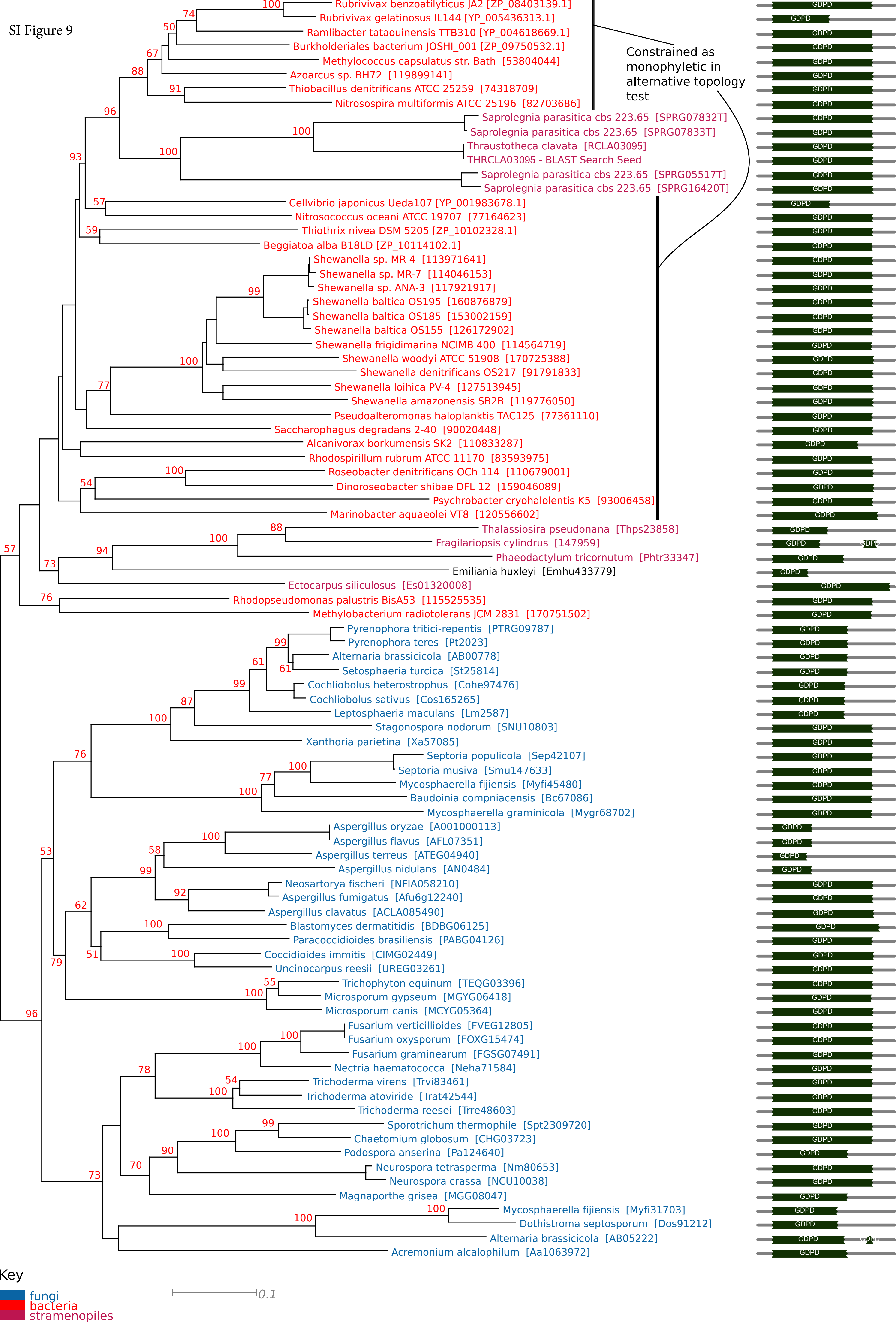

Rubrivivax benzoatilyticus JA2 [ZP_08403139.1]
Rubrivivax gelatinosus IL144 [YP_005436313.1]
Ramlibacter tataouinensis TTB310 [YP_004618669.1]
Burkholderiales bacterium JOSHI_001 [ZP_09750532.1]
Methylococcus capsulatus str. Bath [53804044]
Azoarcus sp. BH72 [119899141]
Thiobacillus denitrificans ATCC 25259 [74318709]
Nitrosospira multiformis ATCC 25196 [82703686]
Saprolegnia parasitica cbs 223.65 [SPRG07832T]
Saprolegnia parasitica cbs 223.65 [SPRG07833T]
Thraustotheca clavata [RCLA03095]
THRCLA03095 - BLAST Search Seed
Saprolegnia parasitica cbs 223.65 [SPRG05517T]
Saprolegnia parasitica cbs 223.65 [SPRG16420T]
Cellvibrio japonicus Ueda107 [YP_001983678.1]
Nitrosococcus oceani ATCC 19707 [77164623]
Thiothrix nivea DSM 5205 [ZP_10102328.1]
Beggiatoa alba B18LD [ZP_10114102.1]
Shewanella sp. MR-4 [113971641]
Shewanella sp. MR-7 [114046153]
Shewanella sp. ANA-3 [117921917]
Shewanella baltica OS195 [160876879]
Shewanella baltica OS185 [153002159]
Shewanella baltica OS155 [126172902]
Shewanella frigidimarina NCIMB 400 [114564719]
Shewanella woodyi ATCC 51908 [170725388]
Shewanella denitrificans OS217 [91791833]
Shewanella loihica PV-4 [127513945]
Shewanella amazonensis SB2B [119776050]
Pseudoalteromonas haloplanktis TAC125 [77361110]
Saccharophagus degradans 2-40 [90020448]
Alcanivorax borkumensis SK2 [110833287]
Rhodospirillum rubrum ATCC 11170 [83593975]
Roseobacter denitrificans OCh 114 [110679001]
Dinoroseobacter shibae DFL 12 [159046089]
Psychrobacter cryohalolentis K5 [93006458]
Marinobacter aquaeolei VT8 [120556602]
Thalassiosira pseudonana [Thps23858]
Fragilariopsis cylindrus [147959]
Phaeodactylum tricornutum [Phtr33347]
Emiliania huxleyi [Emhu433779]
Ectocarpus siliculosus [Es01320008]
Rhodopseudomonas palustris BisA53 [115525535]
Methylobacterium radiotolerans JCM 2831 [170751502]
Pyrenophora tritici-repentis [PTRG09787]
Pyrenophora teres [Pt2023]
Alternaria brassicicola [AB00778]
Setosphaeria turcica [St25814]
Cochliobolus heterostrophus [Cohe97476]
Cochliobolus sativus [Cos165265]
Leptosphaeria maculans [Lm2587]
Stagonospora nodorum [SNU10803]
Xanthoria parietina [Xa57085]
Septoria populicola [Sep42107]
Septoria musiva [Smu147633]
Mycosphaerella fijiensis [Myfi45480]
Baudoinia compniacensis [Bc67086]
Mycosphaerella graminicola [Mygr68702]
Aspergillus oryzae [A001000113]
Aspergillus flavus [AFL07351]
Aspergillus terreus [ATEG04940]
Aspergillus nidulans [AN0484]
Neosartorya fischeri [NFIA058210]
Aspergillus fumigatus [Afu6g12240]
Aspergillus clavatus [ACLA085490]
Blastomyces dermatitidis [BDBG06125]
Paracoccidioides brasiliensis [PABG04126]
Coccidioides immitis [CIMG02449]
Uncinocarpus reesii [UREG03261]
Trichophyton equinum [TEQG03396]
Microsporum gypseum [MGYG06418]
Microsporum canis [MCYG05364]
Fusarium verticillioides [FVEG12805]
Fusarium oxysporum [FOXG15474]
Fusarium graminearum [FGSG07491]
Nectria haematococca [Neha71584]
Trichoderma virens [Trvi83461]
Trichoderma atoviride [Trat42544]
Trichoderma reesei [Tre48603]
Sporotrichum thermophile [Spt2309720]
Chaetomium globosum [CHG03723]
Podospora anserina [Pa124640]
Neurospora tetrasperma [Nm80653]
Neurospora crassa [NCU10038]
Magnaporthe grisea [MGG08047]
Mycosphaerella fijiensis [Myfi31703]
Dothistroma septosporum [Dos91212]
Alternaria brassicicola [AB05222]
Acremonium alcalophilum [Aa1063972]
Constrained as monophyletic in alternative topology test
GDPD
Key
fungi
bacteria
stramenopiles
0.1

SI Figure 10

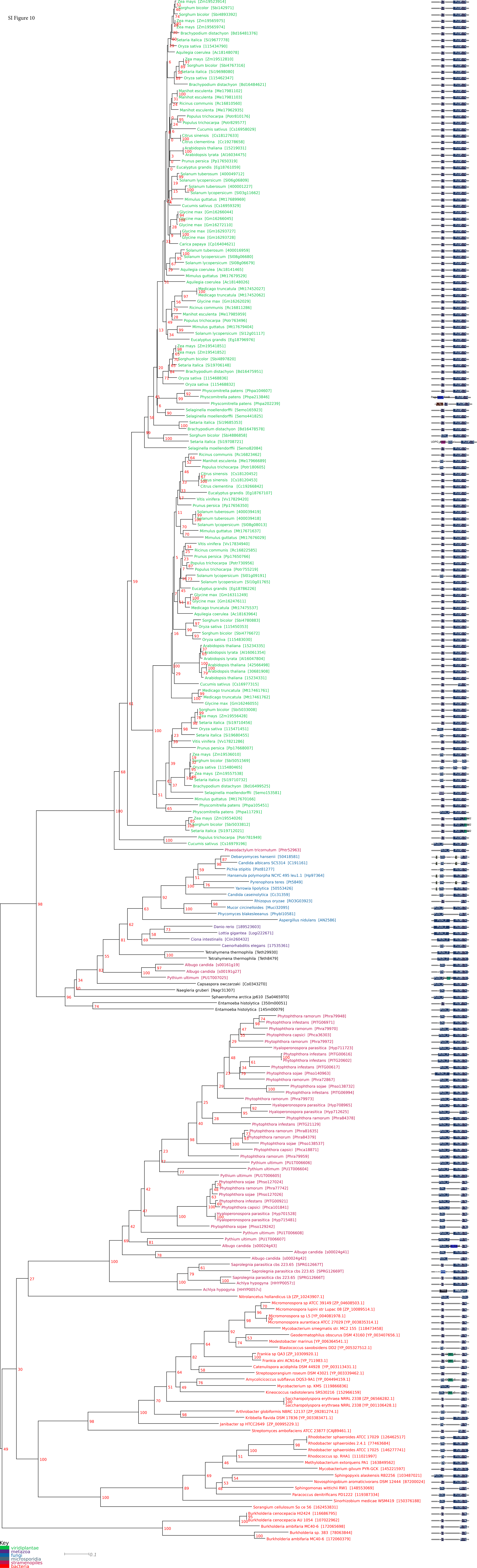
Key
viridiplantae
metazoa
fungi
microsporidia
stramenopiles
bacteria
0.1

SI Figure 11

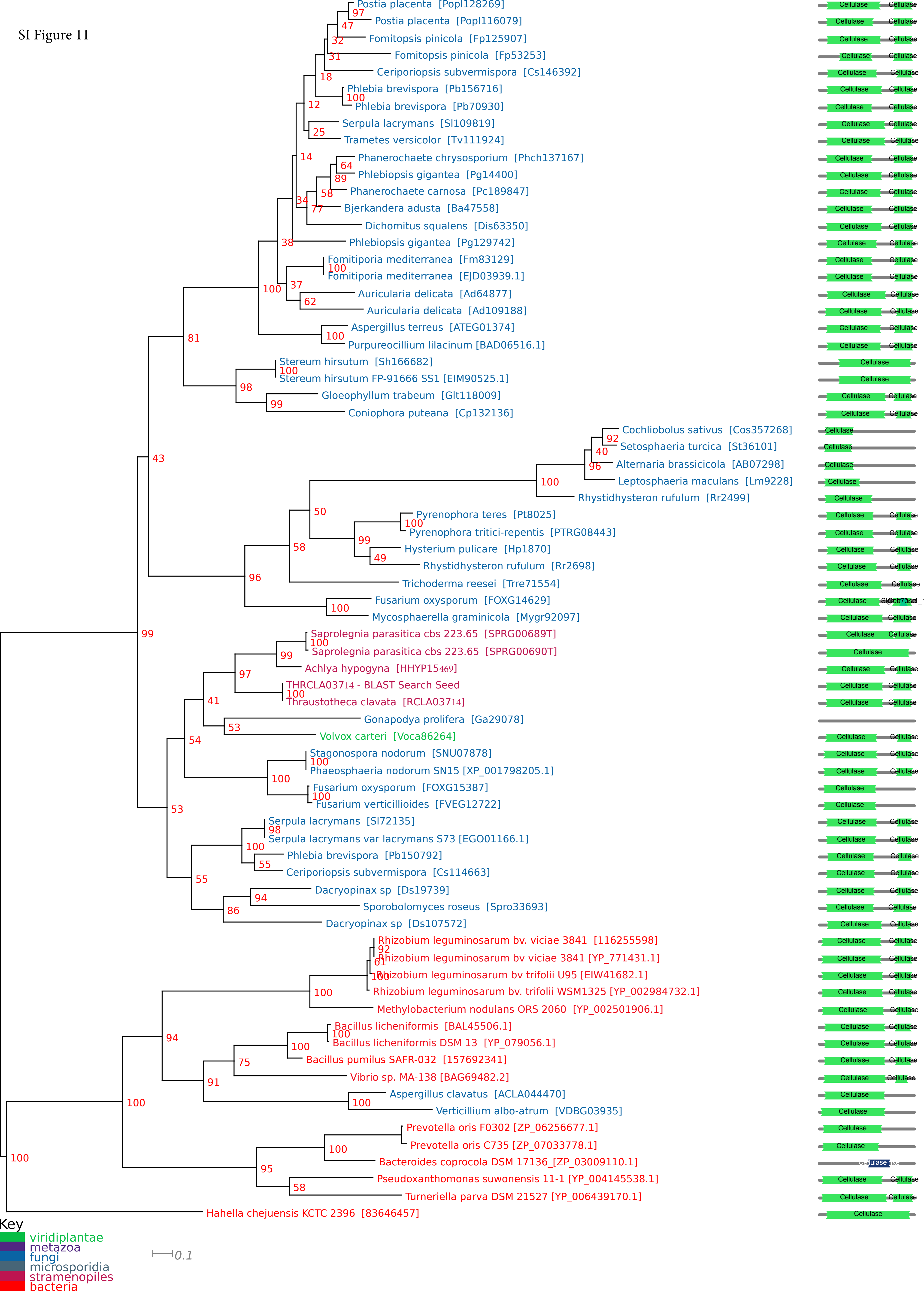

Postia placenta [Popl128269]
Postia placenta [Popl116079]
Fomitopsis pinicola [Fp125907]
Fomitopsis pinicola [Fp53253]
Ceriporiopsis subvermispora [Cs146392]
Phlebia brevispora [Pb156716]
Phlebia brevispora [Pb70930]
Serpula lacrymans [Sl109819]
Trametes versicolor [Tv111924]
Phanerochaete chrysosporium [Phch137167]
Phlebiopsis gigantea [Pg14400]
Phanerochaete carnosa [Pc189847]
Bjerkandera adusta [Ba47558]
Dichomitus squalens [Dis63350]
Phlebiopsis gigantea [Pg129742]
Fomitiporia mediterranea [Fm83129]
Fomitiporia mediterranea [EJD03939.1]
Auricularia delicata [Ad64877]
Auricularia delicata [Ad109188]
Aspergillus terreus [ATEG01374]
Purpureocillium lilacinum [BAD06516.1]
Stereum hirsutum [Sh166682]
Stereum hirsutum FP-91666 SS1 [EIM90525.1]
Gloeophyllum trabeum [Glt118009]
Coniophora puteana [Cp132136]
Cochliobolus sativus [Cos357268]
Setosphaeria turcica [St36101]
Alternaria brassicicola [AB07298]
Leptosphaeria maculans [Lm9228]
Rhystidhysteron rufulum [Rr2499]
Pyrenophora teres [Pt8025]
Pyrenophora tritici-repentis [PTRG08443]
Hysterium pulicare [Hp1870]
Rhystidhysteron rufulum [Rr2698]
Trichoderma reesei [Trre71554]
Fusarium oxysporum [FOXG14629]
Mycosphaerella graminicola [Mygr92097]
Saprolegnia parasitica cbs 223.65 [SPRG00689T]
Saprolegnia parasitica cbs 223.65 [SPRG00690T]
Achlya hypogyna [HHYP15469]
THRCLA03714 - BLAST Search Seed
Thraustotheca clavata [RCLA03714]
Gonapodya prolifera [Ga29078]
Volvox carteri [Voca86264]
Stagonospora nodorum [SNU07878]
Phaeosphaeria nodorum SN15 [XP_001798205.1]
Fusarium oxysporum [FOXG15387]
Fusarium verticillioides [FVEG12722]
Serpula lacrymans [Sl72135]
Serpula lacrymans var lacrymans S73 [EGO01166.1]
Phlebia brevispora [Pb150792]
Ceriporiopsis subvermispora [Cs114663]
Dacryopinax sp [Ds19739]
Sporobolomyces roseus [Spro33693]
Dacryopinax sp [Ds107572]
Rhizobium leguminosarum bv. viciae 3841 [116255598]
Rhizobium leguminosarum bv viciae 3841 [YP_771431.1]
Rhizobium leguminosarum bv trifolii U95 [EIW41682.1]
Rhizobium leguminosarum bv. trifolii WSM1325 [YP_002984732.1]
Methylobacterium nodulans ORS 2060 [YP_002501906.1]
Bacillus licheniformis [BAL45506.1]
Bacillus licheniformis DSM 13 [YP_079056.1]
Bacillus pumilus SAFR-032 [157692341]
Vibrio sp. MA-138 [BAG69482.2]
Aspergillus clavatus [ACLA044470]
Verticillium albo-atrum [VDBG03935]
Prevotella oris F0302 [ZP_06256677.1]
Prevotella oris C735 [ZP_07033778.1]
Bacteroides coprocola DSM 17136_[ZP_03009110.1]
Pseudoxanthomonas suwonensis 11-1 [YP_004145538.1]
Turneriella parva DSM 21527 [YP_006439170.1]
Hahella chejuensis KCTC 2396 [83646457]
Key
viridiplantae
metazoa
fungi
microsporidia
stramenopiles
bacteria
0.1

SI Figure 12

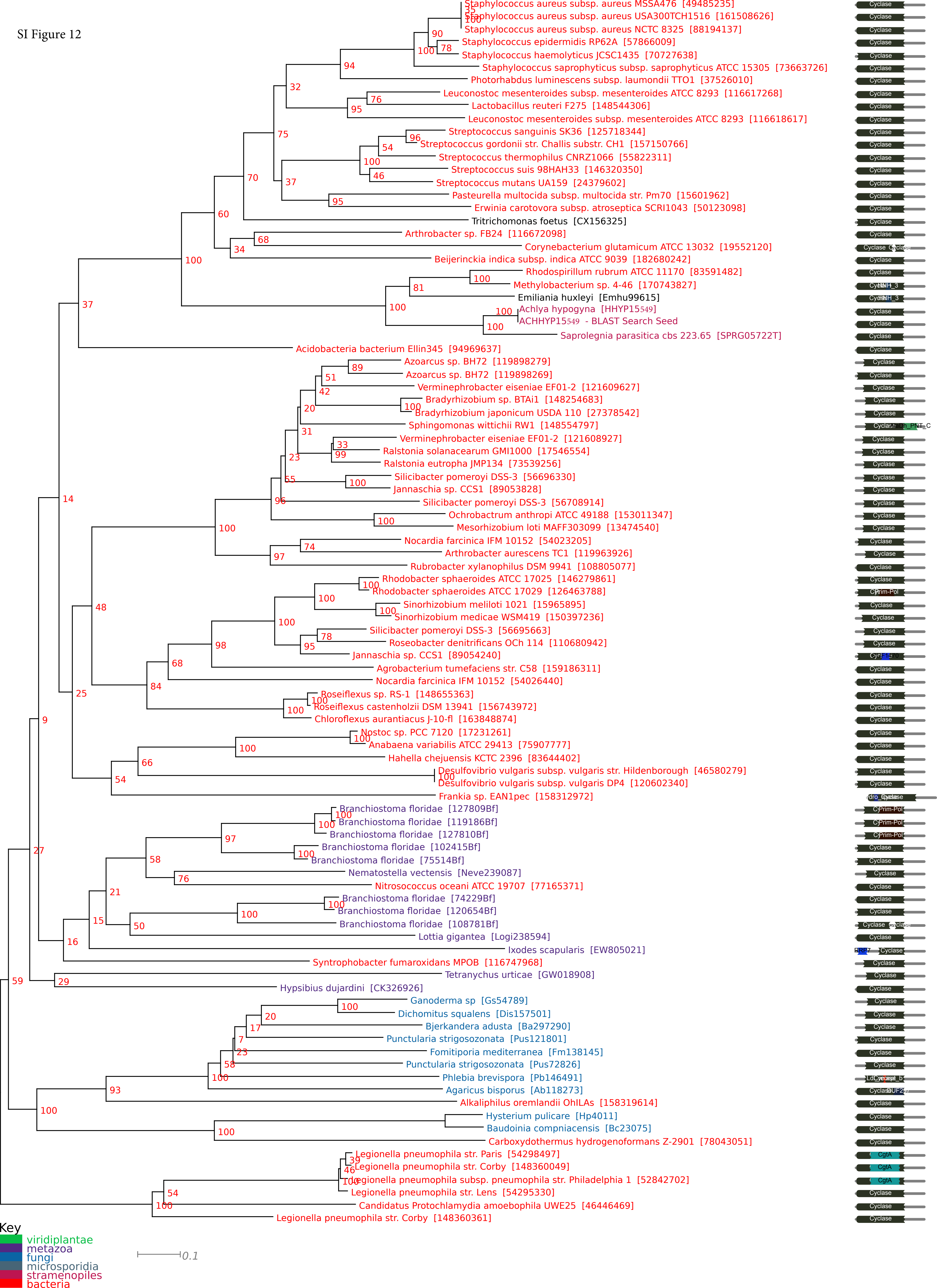

Staphylococcus aureus subsp. aureus MSSA476 [49485235]
Staphylococcus aureus subsp. aureus USA300TCH1516 [161508626]
Staphylococcus aureus subsp. aureus NCTC 8325 [88194137]
Staphylococcus epidermidis RP62A [57866009]
Staphylococcus haemolyticus JCSC1435 [70727638]
Staphylococcus saprophyticus subsp. saprophyticus ATCC 15305 [73663726]
Photorhabdus luminescens subsp. laumondii TTO1 [37526010]
Leuconostoc mesenteroides subsp. mesenteroides ATCC 8293 [116617268]
Lactobacillus reuteri F275 [148544306]
Leuconostoc mesenteroides subsp. mesenteroides ATCC 8293 [116618617]
Streptococcus sanguinis SK36 [125718344]
Streptococcus gordonii str. Challis substr. CH1 [157150766]
Streptococcus thermophilus CNRZ1066 [55822311]
Streptococcus suis 98HAH33 [146320350]
Streptococcus mutans UA159 [24379602]
Pasteurella multocida subsp. multocida str. Pm70 [15601962]
Erwinia carotovora subsp. atroseptica SCRI1043 [50123098]
Tritrichomonas foetus [CX156325]
Arthrobacter sp. FB24 [116672098]
Corynebacterium glutamicum ATCC 13032 [19552120]
Beijerinckia indica subsp. indica ATCC 9039 [182680242]
Rhodospirillum rubrum ATCC 11170 [83591482]
Methylobacterium sp. 4-46 [170743827]
Emiliania huxleyi [Emhu99615]
Achlya hypogyna [HHYP15549]
ACHHYP15549 - BLAST Search Seed
Saprolegnia parasitica cbs 223.65 [SPRG05722T]
Acidobacteria bacterium Ellin345 [94969637]
Azoarcus sp. BH72 [119898279]
Azoarcus sp. BH72 [119898269]
Verminephrobacter eiseniae EF01-2 [121609627]
Bradyrhizobium sp. BTAi1 [148254683]
Bradyrhizobium japonicum USDA 110 [27378542]
Sphingomonas wittichii RW1 [148554797]
Verminephrobacter eiseniae EF01-2 [121608927]
Ralstonia solanacearum GMI1000 [17546554]
Ralstonia eutropha JMP134 [73539256]
Silicibacter pomeroyi DSS-3 [56696330]
Jannaschia sp. CCS1 [89053828]
Silicibacter pomeroyi DSS-3 [56708914]
Ochrobactrum anthropi ATCC 49188 [153011347]
Mesorhizobium loti MAFF303099 [13474540]
Nocardia farcinica IFM 10152 [54023205]
Arthrobacter aurescens TC1 [119963926]
Rubrobacter xylanophilus DSM 9941 [108805077]
Rhodobacter sphaeroides ATCC 17025 [146279861]
Rhodobacter sphaeroides ATCC 17029 [126463788]
Sinorhizobium meliloti 1021 [15965895]
Sinorhizobium medicae WSM419 [150397236]
Silicibacter pomeroyi DSS-3 [56695663]
Roseobacter denitrificans OCh 114 [110680942]
Jannaschia sp. CCS1 [89054240]
Agrobacterium tumefaciens str. C58 [159186311]
Nocardia farcinica IFM 10152 [54026440]
Roseiflexus sp. RS-1 [148655363]
Roseiflexus castenholzii DSM 13941 [156743972]
Chloroflexus aurantiacus J-10-fl [163848874]
Nostoc sp. PCC 7120 [17231261]
Anabaena variabilis ATCC 29413 [75907777]
Hahella chejuensis KCTC 2396 [83644402]
Desulfovibrio vulgaris subsp. vulgaris str. Hildenborough [46580279]
Desulfovibrio vulgaris subsp. vulgaris DP4 [120602340]
Frankia sp. EAN1pec [158312972]
Branchiostoma floridae [127809Bf]
Branchiostoma floridae [119186Bf]
Branchiostoma floridae [127810Bf]
Branchiostoma floridae [102415Bf]
Branchiostoma floridae [75514Bf]
Nematostella vectensis [Neve239087]
Nitrosococcus oceani ATCC 19707 [77165371]
Branchiostoma floridae [74229Bf]
Branchiostoma floridae [120654Bf]
Branchiostoma floridae [108781Bf]
Lottia gigantea [Logi238594]
Ixodes scapularis [EW805021]
Syntrophobacter fumaroxidans MPOB [116747968]
Tetranychus urticae [GW018908]
Hypsibius dujardini [CK326926]
Ganoderma sp [Gs54789]
Dichomitus squalens [Dis157501]
Bjerkandera adusta [Ba297290]
Punctularia strigosozonata [Pus121801]
Fomitiporia mediterranea [Fm138145]
Punctularia strigosozonata [Pus72826]
Phlebia brevispora [Pb146491]
Agaricus bisporus [Ab118273]
Alkaliphilus oremlandii OhILAs [158319614]
Hysterium pulicare [Hp4011]
Baudoinia compniacensis [Bc23075]
Carboxydothermus hydrogenoformans Z-2901 [78043051]
Legionella pneumophila str. Paris [54298497]
Legionella pneumophila str. Corby [148360049]
Legionella pneumophila subsp. pneumophila str. Philadelphia 1 [52842702]
Legionella pneumophila str. Lens [54295330]
Candidatus Protochlamydia amoebophila UWE25 [46446469]
Legionella pneumophila str. Corby [148360361]
Cyclase
CgtA
Prim-Pol
HNH_3
Key
viridiplantae
metazoa
fungi
microsporidia
stramenopiles
bacteria
0.1

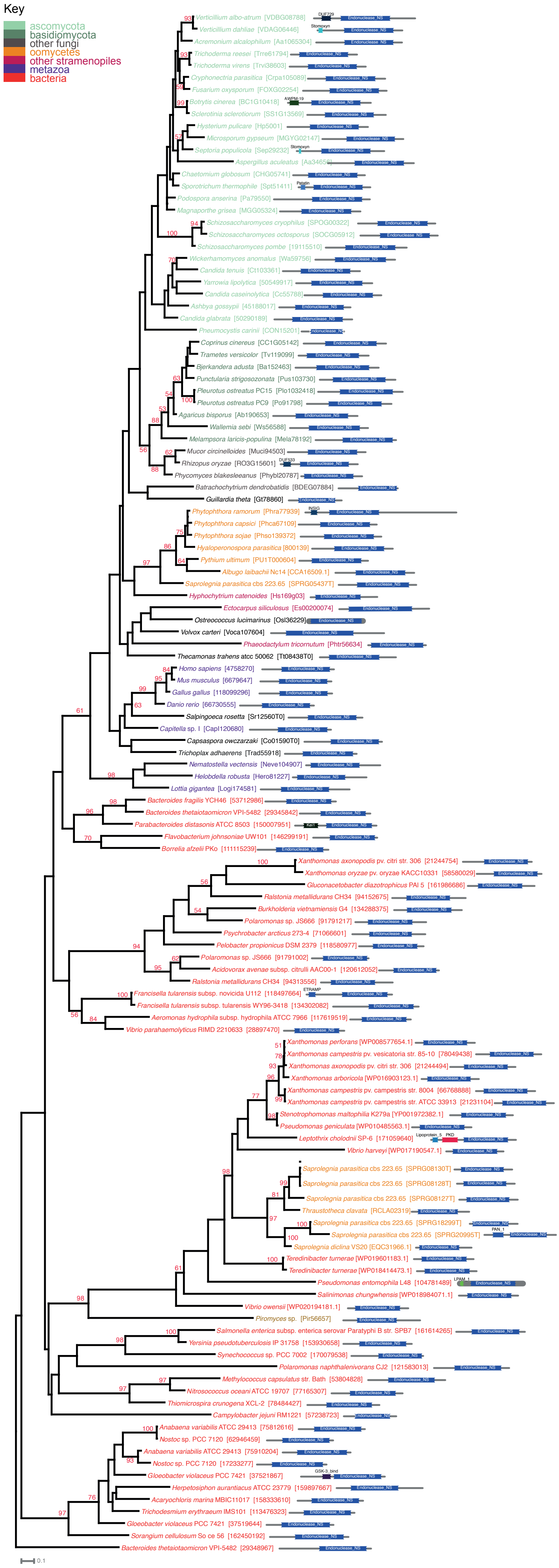
Key
ascomycota
basidiomycota
other fungi
oomycetes
other stramenopiles
metazoa
bacteria
Verticillium albo-atrum [VDBG08788]
Verticillium dahliae [VDAG06446]
Acremonium alcalophilum [Aa1065304]
Trichoderma reesei [Trre61794]
Trichoderma virens [Trvi38603]
Cryphonectria parasitica [Crpa105089]
Fusarium oxysporum [FOXG02254]
Botrytis cinerea [BC1G10418]
Sclerotinia sclerotiorum [SS1G13569]
Hysterium pulicare [Hp5001]
Microsporum gypseum [MGYG02147]
Septoria populicola [Sep29232]
Aspergillus aculeatus [Aa34656]
Chaetomium globosum [CHG05741]
Sporotrichum thermophile [Spt51411]
Podospora anserina [Pa79550]
Magnaporthe grisea [MGG05324]
Schizosaccharomyces cryophilus [SPOG00322]
Schizosaccharomyces octosporus [SOCG05912]
Schizosaccharomyces pombe [19115510]
Wickerhamomyces anomalus [Wa59756]
Candida tenuis [Ct103361]
Yarrowia lipolytica [50549917]
Candida caseinolytica [Cc55788]
Ashbya gossypii [45188017]
Candida glabrata [50290189]
Pneumocystis carinii [CON15201]
Coprinus cinereus [CC1G05142]
Trametes versicolor [Tv119099]
Bjerkandera adusta [Ba152463]
Punctularia strigosozonata [Pus103730]
Pleurotus ostreatus PC15 [Plo1032418]
Pleurotus ostreatus PC9 [Po91798]
Agaricus bisporus [Ab190653]
Wallemia sebi [Ws56588]
Melampsora laricis-populina [Mela78192]
Mucor circinelloides [Muci94503]
Rhizopus oryzae [RO3G15601]
Phycomyces blakesleeanus [Phybl20787]
Batrachochytrium dendrobatidis [BDEG07884]
Guillardia theta [Gt78860]
Phytophthora ramorum [Phra77939]
Phytophthora capsici [Phca67109]
Phytophthora sojae [Phso139372]
Hyaloperonospora parasitica [800139]
Pythium ultimum [PU1T000604]
Albugo laibachii Nc14 [CCA16509.1]
Saprolegnia parasitica cbs 223.65 [SPRG05437T]
Hyphochytrium catenoides [Hs169g03]
Ectocarpus siliculosus [Es00200074]
Ostreococcus lucimarinus [Osl36229]
Volvox carteri [Voca107604]
Phaeodactylum tricornutum [Phtr56634]
Thecamonas trahens atcc 50062 [Tt08438T0]
Homo sapiens [4758270]
Mus musculus [6679647]
Gallus gallus [118099296]
Danio rerio [66730555]
Salpingoeca rosetta [Sr12560T0]
Capitella sp. I [CapI120680]
Capsaspora owczarzaki [Co01590T0]
Trichoplax adhaerens [Trad55918]
Nematostella vectensis [Neve104907]
Helobdella robusta [Hero81227]
Lottia gigantea [Logi174581]
Bacteroides fragilis YCH46 [53712986]
Bacteroides thetaiotaomicron VPI-5482 [29345842]
Parabacteroides distasonis ATCC 8503 [150007951]
Flavobacterium johnsoniae UW101 [146299191]
Borrelia afzelii PKo [111115239]
Xanthomonas axonopodis pv. citri str. 306 [21244754]
Xanthomonas oryzae pv. oryzae KACC10331 [58580029]
Gluconacetobacter diazotrophicus PAl 5 [161986686]
Ralstonia metallidurans CH34 [94152675]
Burkholderia vietnamiensis G4 [134288375]
Polaromonas sp. JS666 [91791217]
Psychrobacter arcticus 273-4 [71066601]
Pelobacter propionicus DSM 2379 [118580977]
Polaromonas sp. JS666 [91791002]
Acidovorax avenae subsp. citrulli AAC00-1 [120612052]
Ralstonia metallidurans CH34 [94313556]
Francisella tularensis subsp. novicida U112 [118497664]
Francisella tularensis subsp. tularensis WY96-3418 [134302082]
Aeromonas hydrophila subsp. hydrophila ATCC 7966 [117619519]
Vibrio parahaemolyticus RIMD 2210633 [28897470]
Xanthomonas perforans [WP008577654.1]
Xanthomonas campestris pv. vesicatoria str. 85-10 [78049438]
Xanthomonas axonopodis pv. citri str. 306 [21244494]
Xanthomonas arboricola [WP016903123.1]
Xanthomonas campestris pv. campestris str. 8004 [66768888]
Xanthomonas campestris pv. campestris str. ATCC 33913 [21231104]
Stenotrophomonas maltophilia K279a [YP001972382.1]
Pseudomonas geniculata [WP010485563.1]
Leptothrix cholodnii SP-6 [171059640]
Vibrio harveyi [WP017190547.1]
Saprolegnia parasitica cbs 223.65 [SPRG08130T]
Saprolegnia parasitica cbs 223.65 [SPRG08128T]
Saprolegnia parasitica cbs 223.65 [SPRG08127T]
Thraustotheca clavata [RCLA02319]
Saprolegnia parasitica cbs 223.65 [SPRG18299T]
Saprolegnia parasitica cbs 223.65 [SPRG20995T]
Saprolegnia diclina VS20 [EQC31966.1]
Teredinibacter turnerae [WP019601183.1]
Teredinibacter turnerae [WP018414473.1]
Pseudomonas entomophila L48 [104781489]
Salinimonas chungwhensis [WP018984071.1]
Vibrio owensii [WP020194181.1]
Piromyces sp. [Pir56657]
Salmonella enterica subsp. enterica serovar Paratyphi B str. SPB7 [161614265]
Yersinia pseudotuberculosis IP 31758 [153930658]
Synechococcus sp. PCC 7002 [170079538]
Polaromonas naphthalenivorans CJ2 [121583013]
Methylococcus capsulatus str. Bath [53804828]
Nitrosococcus oceani ATCC 19707 [77165307]
Thiomicrospira crunogena XCL-2 [78484427]
Campylobacter jejuni RM1221 [57238723]
Anabaena variabilis ATCC 29413 [75812616]
Nostoc sp. PCC 7120 [62946459]
Anabaena variabilis ATCC 29413 [75910204]
Nostoc sp. PCC 7120 [17233277]
Gloeobacter violaceus PCC 7421 [37521867]
Herpetosiphon aurantiacus ATCC 23779 [159897667]
Acaryochloris marina MBIC11017 [158333610]
Trichodesmium erythraeum IMS101 [113476323]
Gloeobacter violaceus PCC 7421 [37519644]
Sorangium cellulosum So ce 56 [162450192]
Bacteroides thetaiotaomicron VPI-5482 [29348967]
0.1
