# Supplementary material for: The Secreted Proteins of Achlya hypogyna and Thraustotheca clavata Identify the Ancestral Oomycete Secretome and Reveal Gene Acquisitions by Horizontal Gene Transfer
Source: Genome Biol Evol. 2014 Dec 18;7(1):120–35. doi: 10.1093/gbe/evu276 (PMC4316629; doi:10.1093/gbe/evu276)
Supplement: Supplementary Data [file supp_evu276_New_Microsoft_Office_Word_Document.docx]

**SI Figures**

**SI Figure 1. Representative Multi-gene alignment of elicitin-like genes in OG5_133826.** Cysteine residues necessary for disulphide bridges are indicated by (*) and bars connect bridging pairs. Red blocks highlight missing cysteine residues. Proteins with a corresponding RNAseq transcript sequence are marked with an “^e^”. ACHHYP = *Achlya hypogyna;* THRCLA = *Thraustotheca clavata;* SPRG = *Saprolegnia parasitica;* AC2VRR = *Albugo candida;* PITG = *Phytophthora infestans*; PYU1= *Pythium ultimum*.

**SI Figure 2. Four multi-gene alignments of elicitin-like genes in OG5_173373, OG5_184829, OG5_184830, and OG5_244191**. Cysteine residues necessary for disulphide bridges are indicated by (*) and bars connect bridging pairs. Red blocks highlight missing cysteine residues. Each of the four elicitin-like gene families has proper folding in the Saprolegnialeans but members of the Peronosporaleans lack the required residues. Proteins with a corresponding RNAseq transcript sequence are marked with an “^e^”. ACHHYP = *Achlya hypogyna;* THRCLA = *Thraustotheca clavata;* SPRG = *Saprolegnia parasitica;* AC2VRR = *Albugo candida;* PITG = *Phytophthora infestans*; PYU1= *Pythium ultimum*.

**SI Figure 3. ML phylogeny of GH18 family proteins.** The phylogeny of GH18 shows HGT to the Saprolegnialeans from a bacterial donor lineages. Oomycetes branch within a gama proteobacterial cluster, within a wider cluster of bacteria. This relationship is supported by strong bootstrap values and alternative topology tests. Bootstrap support values >50 are included at the nodes. PFAM domain structure of each protein is on the right of the sequence ID.

**SI Figure 4. The ML phylogeny of pectate lyase suggests transfer from fungi.** A phylogeny of pectate lyase shows potential HGT from the fungi to the oomycetes, as previously described by Richards et al (2011). In this case HGT is primarily supported by the taxonomic distribution of the gene family rather than the tree topology and support. PFAM domain structure of each protein is on the right of the sequence ID and Bootstrap values >50 are included for each node.

**SI Figure 5. Endoglucnase phylogeny suggests HGT via bacteria. The** ML phylogeny of an endoglucanase, showing potential HGT from bacterial lineages to the oomycetes. The case for HGT is primarily supported by the taxonomic distribution of the gene family rather than the tree topology. Bootstrap support values >50 are shown at each node. PFAM domain structure of each protein is on the right of the sequence ID.

**SI Figure 6. Carbohydrate-binding protein phylogeny suggests HGT via fungi.** ML phylogeny of a carbohydrate-binding protein, showing HGT from the fungi to the oomycetes with Bootstrap support values >50 are shown at each node. HGT is primarily supported by the taxonomic distribution of the gene family rather than the tree topology. PFAM domain structure of each protein is on the right of the sequence ID.

**SI Figure 7. Hydrolase protein phylogeny indicates HGT via bacteria.** ML phylogeny of a hydrolase protein showing HGT from bacterial lineages to the Saprolegnialeans. The oomycete sequences are nested deeply among bacterial sequences, with moderate support. However, the AU test indicates alternative topologies are significantly worse. Bootstrap support values >50 are included at nodes. PFAM domain structure of each protein is on the right of the sequence ID.

**SI Figure 8. GH62 protein phylogeny indicates HGT via fungi.** ML phylogeny of the GH62 protein, showing HGT from the fungi to the Saprolegnialeans. *Thraustotheca clavata* is not represented in this phylogeny. The AU test could not exclude the possibility of an alternative topology where oomycetes branch outside the local fungal clade with the proteins from *Sorangium cellulosum*. Bootstrap support values >50 are included at nodes. PFAM domain structure of each protein is on the right of the sequence ID.

**SI Figure 9. Glycerophosphoryl diester phosphodiesterase** **protein phylogeny indicates HGT via bacteria.** ML phylogeny of glycerophosphoryl diester phosphodiesterase (GDPD) protein, showing HGT from bacterial lineages to the Saprolegnialeans. The nodes supporting the sister relationship between oomycetes and several bacterial lineages are well supported and the AU test indicates alternative topologies are significantly worse. Additional stramenopile taxa also appear in the tree among bacterial taxa, but in positions lacking phylogenetic support. Bootstrap support values >50 are included at nodes. PFAM domain structure of each protein is on the right of the sequence ID.

**SI Figure 10. Phospholipase D indicates HGT via bacteria.** ML tree of phospholipase D showing potential HGT from bacterial lineages to the oomycetes. The majority of oomycete sequences cluster with bacteria, but with low support values. A second oomycete clade, including two *Al. candida* and one *Py. ultimum* sequence is resolved in a well supported clade that includes a broad spectrum of eukaryotes. Bootstrap support values >50 are included at nodes. PFAM domain structure of each protein is on the right of the sequence ID.

**SI Figure 11. GH5 indicates HGT via fungi.** ML tree of GH5 proteins showing potential HGT from the fungi to the Saprolegnialeans. Sequences from all three saprolegnian taxa branch within a well-supported fungal clade, but the inclusion of the green alga, *Volvox carteri,* indicates either a second transfer or a more complex evolutionary history of this protein. Bootstrap support values >50 are included at nodes. PFAM domain structure of each protein is on the right of the sequence ID.

**SI Figure 12. Cyclase protein ML phylogeny suggests bacterial HGT**. The ML phylogeny of the cyclase protein showing potential HGT from the bacteria to *Ac. hypogyna* and *S. parasitica*. The inclusion of a sequence from the haptophyte, *Emiliania huxleyi*, and the generally low bootstrap support for the clade prevent a more definitive HGT identification. Bootstrap support values >50 are included at the nodes and PFAM domain structure of each protein is on the right of the sequence ID.

**SI Figure 13. The ML phylogeny of the *Saprolegnia parasitica* protein, SPRG_08128.** An ML phylogeny of this protein suggests this is a potential HGT from bacteria into the saprolegnian oomycetes. A second oomycete clade, including a *Sp. parasitica* homolog, clusters among other eukaryotic taxa. Bootstrap support values are the number of trees supported out of 1000. PFAM domain structure of each protein is on the right of the sequence ID.
